# Amniotic fluid cell-free transcriptome: a glimpse into fetal development and placental cellular dynamics during normal pregnancy

**DOI:** 10.1186/s12920-020-0690-5

**Published:** 2020-02-12

**Authors:** Adi L. Tarca, Roberto Romero, Roger Pique-Regi, Percy Pacora, Bogdan Done, Marian Kacerovsky, Gaurav Bhatti, Sunil Jaiman, Sonia S. Hassan, Chaur-Dong Hsu, Nardhy Gomez-Lopez

**Affiliations:** 1 Perinatology Research Branch, Division of Obstetrics and Maternal-Fetal Medicine, Division of Intramural Research, 𝐸𝑢𝑛𝑖𝑐𝑒 𝐾𝑒𝑛𝑛𝑒𝑑𝑦 𝑆ℎ𝑟𝑖𝑣𝑒𝑟 National Institute of Child Health and Human Development, National Institutes of Health, U. S. Department of Health and Human Services (NICHD/NIH/DHHS), Detroit, MI USA; 2 Department of Obstetrics and Gynecology, Wayne State University School of Medicine, Detroit, MI USA; 3 Department of Computer Science, Wayne State University College of Engineering, Detroit, MI USA; 4 Department of Obstetrics and Gynecology, University of Michigan, Ann Arbor, MI USA; 5 Department of Epidemiology and Biostatistics, Michigan State University, East Lansing, MI USA; 6 Center for Molecular Medicine and Genetics, Wayne State University, Detroit, MI USA; 7 Detroit Medical Center, Detroit, MI USA; 8 Department of Pathology, Hutzel Women’s Hospital, Wayne State University School of Medicine, Detroit, MI USA; 9 Department of Physiology, Wayne State University School of Medicine, Detroit, MI USA; 10 Department of Biochemistry, Microbiology and Immunology, Wayne State University School of Medicin, Detroit, MI USA

**Keywords:** Cell-free RNA, Differential splicing, Differential expression, Single-cell genomic signature, Tissue-specific signature, Gestational age, Maternal obesity, Fetal sex

## Abstract

**Background:**

The amniotic fluid (AF) cell-free transcriptome is modulated by physiologic and pathologic processes during pregnancy. AF gene expression changes with advancing gestation reflect fetal development and organ maturation; yet, defining normal expression and splicing patterns for biomarker discovery in obstetrics requires larger heterogeneous cohorts, evaluation of potential confounding factors, and novel analytical approaches.

**Methods:**

Women with a normal pregnancy who had an AF sample collected during midtrimester (*n* = 30) or at term gestation (*n* = 68) were included. Expression profiling at exon level resolution was performed using Human Transcriptome Arrays. Differential expression was based on moderated t-test adjusted *p* < 0.05 and fold change > 1.25; for differential splicing, a splicing index > 2 and adjusted *p* < 0.05 were required. Functional profiling was used to interpret differentially expressed or spliced genes. The expression of tissue-specific and cell-type specific signatures defined by single-cell genomics was quantified and correlated with covariates. *In-silico* validation studies were performed using publicly available datasets.

**Results:**

1) 64,071 genes were detected in AF, with 11% of the coding and 6% of the non-coding genes being differentially expressed between midtrimester and term gestation. Expression changes were highly correlated with those previously reported (R > 0.79, *p* < 0.001) and featured increased expression of genes specific to the trachea, salivary glands, and lung and decreased expression of genes specific to the cardiac myocytes, uterus, and fetal liver, among others. 2) Single-cell RNA-seq signatures of the cytotrophoblast, Hofbauer cells, erythrocytes, monocytes, T and B cells, among others, showed complex patterns of modulation with gestation (adjusted *p* < 0.05). 3) In 17% of the genes detected, we found differential splicing with advancing gestation in genes related to brain development processes and immunity pathways, including some that were missed based on differential expression analysis alone.

**Conclusions:**

This represents the largest AF transcriptomics study in normal pregnancy, reporting for the first time that single-cell genomic signatures can be tracked in the AF and display complex patterns of expression during gestation. We also demonstrate a role for alternative splicing in tissue-identity acquisition, organ development, and immune processes. The results herein may have implications for the development of fetal testing to assess placental function and fetal organ maturity.

## Background

Amniotic fluid (AF) provides mechanical protection to the developing fetus and has important nutritional and immunologic roles [1–3]. Given that AF is in direct contact with the placenta and fetal membranes, surrounding the fetus, while passing through several fetal cavities (e.g. gastrointestinal and respiratory tracts), it is expected that its molecular composition is both reflective of and contributes to fetal wellbeing [4–6].

For decades, AF sampled through transabdominal amniocentesis has been used to assess the fetal karyotype [7–11], lung maturity [12–15], and presence of inflammatory conditions, such as intra-amniotic infection and sterile intra-amniotic inflammation [16–82]. Although the ultimate goal is to develop fetal testing via non-invasive sampling, such as urine or maternal blood, the advantages of AF for biomarker discovery have often been recognized [83–87].

Among the omics platforms used to study AF (see Kamath-Rayne et al. [88] for a review), the analysis of cell-free mRNA (cfRNA) has the advantage of being easier to profile than its proteomics [45, 89, 90] and metabolomics [91–96] counterparts. The AF cfRNAs are thought to be contributed directly by the fetus and by apoptotic amniocytes [97] and have been shown to be altered by physiologic and pathologic factors such as gestational age [83, 85, 98], fetal sex [83], maternal obesity [99], genetic syndromes [100–102], and neonatal co-morbidities [85] (see Zwemer and Bianchi for a review [97]). Of all the factors previously reported to be reflected in the AF transcriptome, advancing gestation seems to have the most dramatic effect on the AF transcriptome considering the number of genes differentially expressed. AF gene expression changes with gestational age have been associated with cell types found in the intrauterine environment and with the development of multiple organ systems [78, 85, 98, 103]. These results point to the possible use of the AF transcriptome to complement fetal lung maturity evaluation [85, 98] when elective delivery prior to term is considered, and also to discover biomarkers for the ‘great obstetrical syndromes’ [104].

Currently, several limitations exist in defining a reference of gene expression and splicing patterns during gestation in normal pregnancy based on publicly available data. Chief among them is the rather limited sample size and use of measurement platforms that do not allow assessment of non-coding RNAs and splicing patterns. Moreover, while previous studies considered multiple functional databases and tissue-specific gene sets to interpret differentially expressed genes in AF [85, 98], detailed signatures of specific cell-types were not readily available. Recently, single-cell genomics studies enabled the assessment of the maternal-fetal crosstalk by quantifying mRNA signatures specific to sub-populations of placental cells [105–107]. These mRNA signatures were shown to be detectable by cell-free [105] and cellular [108] transcriptome analyses of maternal blood; however, to date, they have not been evaluated in AF.

Therefore, the current study aimed i) to evaluate the effect of gestational age on AF cfRNA expression and splicing while considering relevant potential covariates (fetal sex, maternal characteristics, mode of sample collection, and indication for amniocentesis), ii) to determine whether the molecular dynamics of specific placental cell populations, such as those recently described by single-cell RNA sequencing [105], can be tracked by AF cfRNA analysis, and iii) to assess the extent to which previously reported changes in the overall gene expression with gestational age, fetal sex, and maternal obesity can be replicated, given differences in populations and profiling techniques.

## Methods

### Study design

To conduct a prospective longitudinal study, we enrolled pregnant women attending the Center for Advanced Obstetrical Care and Research of the Perinatology Research Branch, *Eunice Kennedy Shriver* National Institute of Child Health and Human Development (NICHD), National Institutes of Health, U.S. Department of Health and Human Services; Wayne State University School of Medicine; and Hutzel Women’s Hospital of the Detroit Medical Center (Detroit, Michigan, USA). Based on this cohort, we designed a retrospective study to include 30 women who underwent transabdominal amniocentesis during the midtrimester at 16.4–24.0 weeks of gestation (median = 21.1 weeks) to assess the fetal karyotype or to rule out intra-amniotic inflammation/infection. In all cases tested, the karyotype was normal. The study also included 68 women at term not in labor (TNL) who had an AF sample collected either by transabdominal amniocentesis (to assess fetal lung maturity) or for research purposes during Cesarean section at 37.1–40.9 weeks of gestation (median = 39.0 weeks). All study participants included herein delivered at term and had a normal singleton pregnancy with appropriate fetal growth, according to the INTERGROWTH-21st birthweight standard [109], which was previously found to match the population of patients attending our research clinic [110]. Five ml of AF were collected from each woman and processed according to the recommended protocol [83].

### RNA extraction

Starting with 5 ml of AF, we applied the Plasma/Serum RNA Purification Maxi Kit (#56200; Norgen Biotek Corp., Thorold, Ontario, Canada), including the optional DNAse treatment, according to the manufacturer’s protocol. Following RNA extraction, each sample was concentrated to a volume of 12 μl by using the RNA Clean & Concentrator-5 Kit (#R1015; Zymo Research, Irvine, California, USA). The concentrates were then quantified by UV spectrophotometry on a DropSense 96 system (PerkinElmer, Waltham, MA, USA) and then quality assessed on the Agilent 2200 TapeStation system (Agilent Technologies, Santa Clara, California, USA).

### Microarray analysis

Ten ng of RNA was reverse-transcribed and amplified using the Affymetrix GeneChip® WT Pico Reagent Kit (Affymetrix, Inc., Santa Clara, California, USA), following the manufacturer’s suggested protocol. Briefly, 5.5 μg of sense-stranded cDNA was fragmented, labeled, and hybridized in a final volume of 200 μl to the Affymetrix GeneChip® Human Transcriptome Array 2.0 in an Affymetrix hybridization oven at 45 °C at 60 rpm for 16 h. Wash and stain steps were performed utilizing an Affymetrix GeneChip® Fluidics Station 450 and scanned on an Affymetrix GeneChip® Scanner 3000. Raw intensity data were generated from array images using Affymetrix GeneChip™ Command Console Software.

### Data analysis

#### Preprocessing

Raw gene expression data of more than 6.0 million microarray probes and 98 microarrays were preprocessed (background correction, normalization, and summarization) using the Robust Multi-array Average (RMA) approach [111] implemented in the *oligo* package [112]. Expression summarization was obtained into one value per sample and transcript cluster (gene level data) for differential expression analysis as well as at the level of each exon or exon-exon junction (probe set level data) for differential splicing analysis. Transcript clusters were assigned to unique genes using annotation from the *hta20transcriptcluster.db* package of *Bioconductor* [113]. Only genes expressed above background (*p* < 0.05) in at least 25% of the samples of either group (midtrimester or TNL) were retained for further differential expression and differential splicing analyses. Detection *p*-values were obtained using the Expression Console, version 1.4. (Affymetrix, Inc.).

#### Differential expression

Gene level log_2_ expression data were analyzed using linear models implemented in the *limma* [114] package in *Bioconductor* [113]. The gestational-age effect (TNL versus midtrimester or early midtrimester versus late midtrimester) was assessed while adjusting only for covariates with significant effect on gene expression among those considered (fetal sex, maternal race, obesity, parity, smoking status, and mode of sample collection). The effect of fetal sex was assessed while adjusting for gestational age. Expression changes were deemed significant based on false discovery rate q-values < 0.05 and a minimum fold change of 1.25-fold, which are known to be rather conservative for this microarray platform [108].

#### Differential splicing

Differential splicing was assessed in relation to gestational age, fetal sex, and obesity based on the splicing index (SI) method [115] implemented in the Transcriptome Analysis Console (TAC) version 4.0 (Affymetrix, Inc.) using *netaffx_release_36* gene annotations. The splicing index represents the difference in average exon usage between groups, where exon usage is defined as exon level expression relative to overall gene abundance in a given sample. An adjusted *p* < 0.05 and SI > 2.0-fold for one exon/exon-exon junction of a gene was required to infer significant alternative splicing for that gene. A second differential splicing algorithm, also based on the SI concept, was applied: *DiffSplice*, implemented in the *limma* package [114], uses an F-test to assess whether log fold changes (between groups) differ among exons of the same gene. Furthermore, based on the SI and corresponding *p*-values, specific patterns of differential splicing were identified among the following: cassette exon, mutually exclusive exons, alternative 5′ sites, alternative 3′ sites, and intron retention (see Blencowe B. for a review [116]), implementing the Exon Event Estimation algorithm in TAC 4.0 software, also used to display differential splicing data for specific genes.

#### Functional profiling

The lists of differentially expressed/spliced genes for each factor considered (e.g. gestational age) were tested for enrichment based on chromosomal location and membership in previously described functional categories and pathways as well as on specificity to tissues and cell types. The functional databases considered were the Developmental FunctionaL Annotation at Tufts (DFLAT) database [117] and the Curated Gene Sets (C2) collection from the Molecular Signatures Database (MSigDB) database [118]. Tissue-specific genes were defined as those with median expression > 30 times higher in a given tissue than the median expression of all other tissues described in the Gene Atlas [119]. This cut-off was chosen to enable the direct comparison of findings to previous reports [120]. All enrichment analyses were based on a hypergeometric test (equivalent to a Fisher’s exact test) and accounted for multiple testing, with q < 0.05 being considered a significant result.

#### Analysis of single-cell RNA-Seq signature expression in AF

Log_2_ microarray expression data were transformed into Z-scores for each gene by subtracting the mean and dividing to the standard deviation calculated from the reference study group (e.g. midtrimester when assessing changes from midtrimester to term). The Z-scores in each sample were averaged over the set of genes previously defined as specific to a given population of cells defined by single-cell RNA-Seq analyses in Tsang et al. [105] The collection of gene sets included those for extravillous trophoblasts, cytotrophoblasts, the syncytiotrophoblast, decidual cells, dendritic cells, endothelial cells, erythrocytes, Hofbauer cells, stromal cells, vascular smooth muscle cells, B cells, T cells, and monocytes. Unlike averaging over the normalized expression of genes, as in Tsang et al. [105], the standardization of expression data ensures that genes contribute equally to the gene set summary [108, 121]. The average Z-score for each single-cell signature was compared between the TNL and midtrimester groups by using the Wilcoxon rank sum test as well as within the midtrimester group via linear models by correlating gene expression with gestational age (continuous).

## Results

### Clinical characteristics of the study population

We profiled the cell-free transcriptome in AF samples collected during gestation at midtrimester (*n* = 30) and at term from women without labor (*n* = 68). The median gestational ages were 21 and 39 weeks in the midtrimester and TNL groups, respectively, at the time of sample collection. Women in the midtrimester group were more likely to be nulliparous (26.7%) compared to those in the TNL group (4.4%) (*p* = 0.003). There were no differences in maternal age, body mass index (BMI), smoking status, fetal sex, and gestational age at delivery between the midtrimester and TNL groups (Table 1).

**Table 1:** Demographic characteristics of the study population. Continuous variables were compared between groups using Welch’s t-test and are summarized as medians (interquartile range). Categorical variables are shown as number (%) and were compared using a Fisher’s exact test

	Midtrimester *n* = 30	TNL *n* = 68	*p*-value
Age	25 (23–29.75)	27 (23–31)	0.302
Body Mass Index	26.2 (24.1–30.3)	28.3 (24–34)	0.462
Parity: 0	8/30 (26.7%)	3/68 (4.4%)	0.003
≥1	22/30 (73.3%)	65/68 (95.6%)	
Race:			
*African American*	26/30 (86.7%)	56/68 (82.4%)	NS
*White*	1/30 (3.3%)	5/68 (7.4%)	
*Hispanic*	2/30 (6.7%)	2/68 (2.9%)	
*Other*	1/30 (3.3%)	5/68 (7.4%)	
Smoking	4/30 (13.3%)	13/68 (19.1%)	0.574
Fetal Sex (*Male*)	13/30 (43.3%)	38/68 (55.9%)	0.279
Sample GA (weeks)	21.05 (19.9–22.6)	39 (38.9–39.3)	< 0.001
Delivery GA (weeks)	38.8 (37.9–39.6)	39 (38.9–39.3)	0.34
Sample collection			
*Transabdominal*	30 (100%)	4/68 (6%)	< 0.001
*Cesarean delivery*	0 (0%)	64/68 (94%)	
Indication for amniocentesis			N/A
*Rule out Infection/ Inflammation*	20 (66.7%)		
*Karyotyping*	12 (40%)^b^		
*Fetal lung maturity*		3/67^a^	

*GA* Gestational age; *TNL* Term not in labor. ^a^ For one case of the four obtained by transabdominal amniocentesis, the indication was not available. ^b^ 3/12 patients with indication for Karyotyping had also an indication for ruling out infection/inflammation

### Factors affecting the AF transcriptome in normal pregnancy

**Fig. 1:**
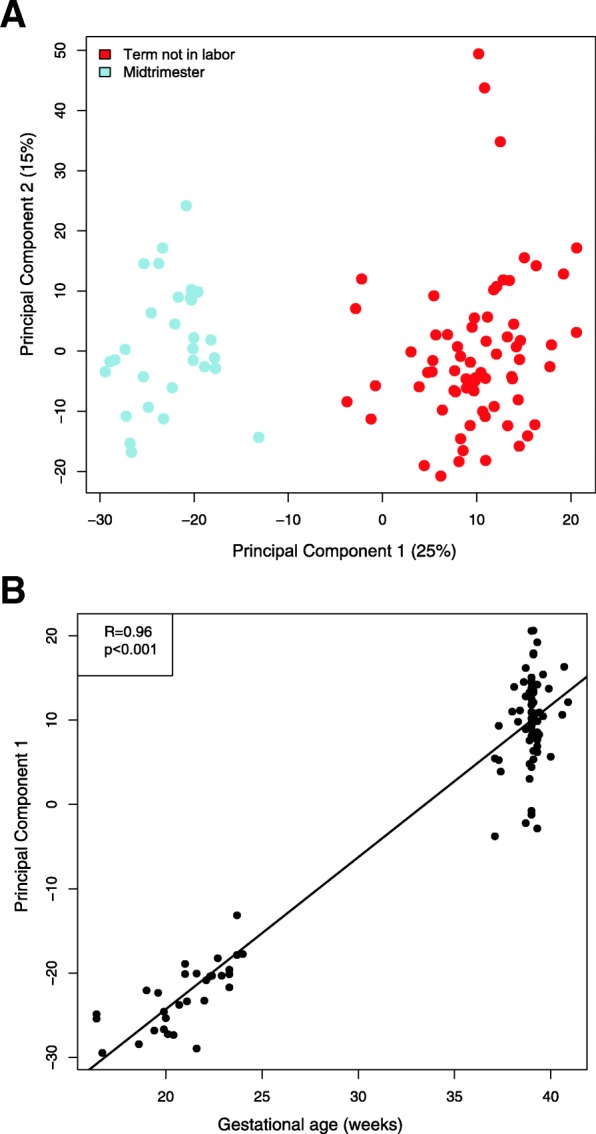
Principal component analysis of amniotic fluid cell-free RNA expression in normal pregnancy. The principal components (PC) were derived from expression of the top 1000 most varying genes (unsupervised selection). The first panel (**a**) depicts each sample based on the first two principal components (PC1 and PC2). The values in parentheses are the % of variance explained by each principal component. TNL: term not in labor. The linear correlation between gestational age and PC1 is also shown in panel (**b**)

The Human Transcriptome Array 2.0 platform used in this study was designed to probe at exon-level resolution the expression of 44,699 protein coding and 22,829 non-protein coding transcript clusters, simply referred to herein as genes (67,528 total). Of these, 64,071 (95%) were deemed expressed (present) in at least 25% of the AF samples in either the midtrimester or the TNL group, and were retained for further differential expression and splicing analyses. An unsupervised principal component analysis representation of the genome-wide gene expression profiles based on the top 1000 most varying genes across all samples is shown in Fig. 1a. The samples in this figure are clustered by gestational age groups, which is suggestive [122] of large between-group differences. Moreover, the first principal component (PC1) was not only linearity correlated with gestational age overall (*R* = 0.96) but also within the subset of midtrimester samples alone (*R* = 0.72) (both, *p* < 0.001) (Fig. 1b).

We considered several maternal and fetal characteristics as well as the mode of AF sample collection and indication for amniocentesis to evaluate their effects on overall gene expression and splicing (Table 2). Overall, gene expression and splicing patterns were found to change dramatically with gestational age and modestly with fetal sex. There was also weak evidence of changes in maternal obesity, whereas other maternal characteristics (parity, smoking status, and race) and mode of AF sample collection (cesarean section versus transabdominal amniocentesis) and indication for amniocentesis did not have a significant effect on overall gene expression (Table 2). The effects of gestational age and fetal sex on gene expression and splicing are detailed in the next section.

**Table 2:** Effect of covariates on amniotic fluid transcriptome. Early midtrimester is defined as gestational age 16.4–21.0 weeks and late midtrimester is defined as gestational age 21.1–24.0 weeks. Differential splicing was assessed only for contrasts with significant differential expression

Effect	Comparison/sample size	Adjustment variable	Diff. expressed Genes	Diff. spliced Genes	In-silico validation
Gestational age effect during midtrimester	Late midtrimester (*n* = 15) vs. Early Midtrimester (*n* = 15)	Fetal sex	413	806	
Gestational age (overall)	TNL (*n* = 68) vs. Midtrimester (*n* = 30)	Fetal sex	6194	8566	Hui et al. 2013 [98].; Kamath-Rayne et al. 2015 [85]
Mode of collection in TNL	Cesarean delivery (*n* = 64) versus transabdominal amniocentesis (*n* = 4)	Fetal sex	0	0	
Smoking	Smoker (*n* = 17) vs. non-smoker (*n* = 79)	Gestational age and fetal sex	0	0	
Fetal sex	Male (*n* = 51) vs. Female (*n* = 47)	Gestational age	252	240	Larrabee et al. 2005 [83]
Maternal race	Caucasian (*n* = 6) vs. African American (*n* = 82)	Gestational age and fetal sex	0	0	
Parity	Parous (*n* = 87) vs. Nulliparous (*n* = 11)	Gestational age and fetal sex	0	0	
Obesity (overall)	Obese (*n* = 38) vs. Lean (*n* = 33)	Gestational age and fetal sex	0	0	Edlow et al. 2014 [99]
Obesity in midtrimester	Obese(*n* = 8) vs. Lean (*n* = 11)	Gestational age and fetal sex	0	0	Edlow et al. 2014 [99]
Indication for amniocentesis during midtrimester*	Detection of infection/inflammation (*n* = 17) vs. assessing fetal karyotype (*n* = 9)	Gestational age and fetal sex	0	0	

*TNL* Term not in labor. Indications for both

### Effect of gestational age on the AF cfRNA

#### Differential expression

Advancing gestational age from midtrimester to term was associated with expression changes in about 10% of the genes detected in AF (6194/64,071), representing 11% of the coding and 6% of the non-coding genes detected. Gene expression changes included both an increase in expression from midtrimester to term gestation (2776 genes) and a decrease (3418 genes) (q-value < 0.05 and fold change > 1.25) (Additional file 7: Table S1). In addition to assessing how the AF transcriptome changes from midtrimester to term, we explored for the first time how the transcriptional program changes with advancing gestation during the midtrimester, which was not feasible in previous studies due to sample size limitations. A differential expression analysis between samples collected at early (16.4–21.0 weeks) and late (21.1–24.0 weeks) midtrimester identified 413 differentially expressed genes (Additional file 8: Table S2).

#### *In-silico* validation of differential expression

**Fig. 2:**
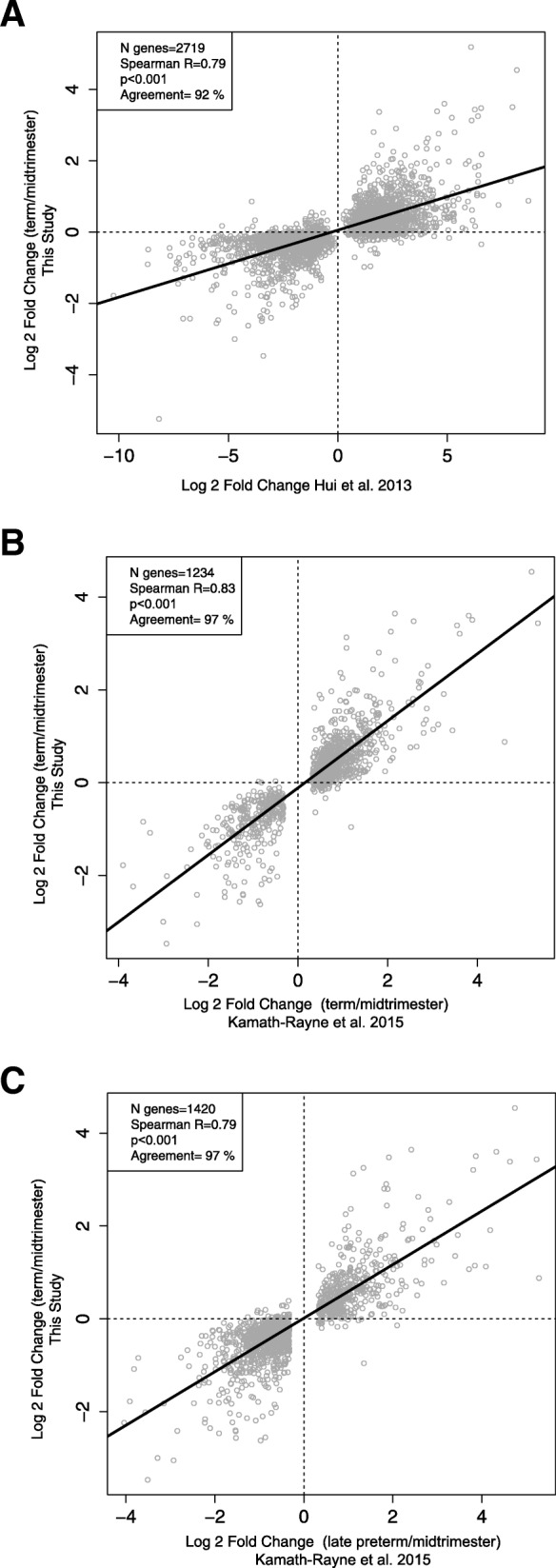
In-silico validation of differential expression between midtrimester and term gestation groups. Each dot represents a unique annotated gene. The y axis represents the log_2_ fold change (term/midtrimester) obtained in the current study. The x-axis represents: **a**) log_2_ fold change reported by Hui et al. [120] (term vs midtrimester); **b**) log_2_ fold change based on a re-analysis of RNA-Seq data reported by Kamath-Rayne et al. [85] between late preterm and midtrimester gestation; and **c**) between term and midtrimester gestation. R: Spearman’s correlation coefficient

To demonstrate the plausibility of AF differential expression from midtrimester to term, we conducted an *in-silico* analysis to determine whether previously reported findings support our data and vice versa. In the first analysis, we considered all 2719 genes reported by Hui et al. [120] to change with gestational age (term versus midtrimester) and they were also detected present in the samples in this study (regardless of statistical significance). We found a substantial agreement in terms of direction of change (92% match) and correlation of log fold changes (Spearman’s correlation, *R* = 79%, *p* < 0.0001) (Fig. 2a). When only the subset of 1332 genes that were significant in both studies were considered, the agreement in terms of direction of change reached 99%, while the correlation of log_2_ fold changes increased to 0.82 (*p* < 0.001). In a second *in-silico* experiment, we considered the genes detected in the current study (regardless of significance) that were differentially expressed (q-value < 0.05 fold change > 1.25) based on a re-analysis of the RNA-Seq data reported by Kamath-Rayne et al. [85]. Our expression-change estimates for the TNL to midtrimester comparison were highly correlated with those of the 1234 genes that differed between late preterm and midtrimester (*R* = 0.83, *p* < 0.001, 97% direction of change agreement, Fig. 2b) and with those of the 1420 genes that differed between term and midtrimester (*R* = 0.79, *p* < 0.001, 97% direction of change agreement, Fig. 2c), obtained by re-analysis of the Kamath-Rayne et al. [85] dataset. Overall, these results demonstrate high cross-study reproducibility of gene-level differential-expression changes with gestation in amniotic fluid.

#### Functional profiling

##### Chromosome enrichment

Although differential expression with advancing gestation was noted throughout the genome, five chromosomes (Chr1, Chr4, Chr6, Chr12, and Chr15) displayed slightly more differential expression than expected by chance (see Additional file 1: Figure S1) (q < 0.05, OR 1.2–1.4 for all).

##### Biological processes enrichment

A wide range of biological processes documented in the *DFLAT* database [117] were over-represented among the genes that change in expression from midterm to term gestation in the AF. Placental development, organ development (lung, liver, brain, heart, etc.), and immune-related pathways (*positive regulation of immune response*, *activation of immune response*, *T-cell activation*) are a few of more than 1500 biological processes related to gene expression changes (see Additional file 9: Table S3).

##### Canonical pathways enrichment

Similarly, about 250 pathways curated in the *MSigDB* collection were associated with gene expression changes with gestational age, such as the KEGG database *systemic lupus erythematosus* pathways and the *Reactome* database *amyloids*, *cell cycle*, *developmental biology*, *immune system*, *cytokine signaling in immune system*, and *mRNA splicing* pathways (q < 0.05) (Additional file 10: Table S4).

##### Tissue enrichment and signature analysis

**Fig. 3:**
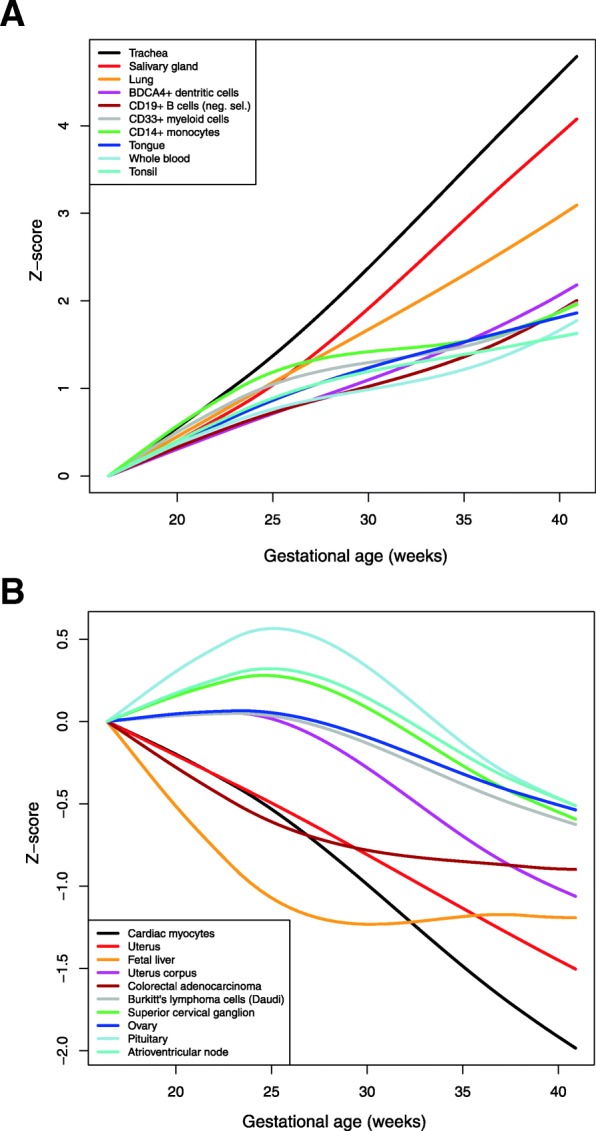
Changes in the expression of tissue-specific signatures with gestational age. For each tissue, the expression of the top 20 most-specific genes (based on the Gene Atlas dataset) was transformed into a Z-score and averaged in each AF sample. A Robust Locally Weighted Regression and Smoothing Scatterplots (LOESS) model fit through the Z-scores as a function of gestational age is shown using lines (see Fig. S2 for individual values). Tissue signature trends are set to have the same value at 16 weeks of gestation. Differentially expressed tissue signatures were sorted by the magnitude of change from 16 to 41 weeks of gestation and the top 10 tissues with increased (**a**) and deceased (**b**) expression are shown. AF, amniotic fluid

Finally, we tested the association of mRNA modulation with advancing gestational age and defined sets based on the Gene Atlas [119] and found that most over-represented organs among genes with higher expression at term were the trachea, lung, salivary glands, tonsils, tongue, colon, bone marrow, skin, and fetal lung, among others listed in Additional file 11: Table S5 (q < 0.05). Although fetal skin was not represented in the Gene Atlas-based analysis, it was proposed by Hui et al. [120] that skin-specific transcripts identified in amniotic fluid are likely derived from the fetal skin. The most-enriched organs for genes with higher expression during midterm gestation were the small intestine, placenta, uterus, and specific cell types (e.g. CD105+ endothelial cells, cardiac myocytes), among others listed in Additional file 12: Table S6 (q < 0.05). These findings were also supported by an alternative analysis in which the expression signature of each tissue type (based on the average of the top 20 most-specific genes) was analyzed as a continuous response as a function of gestational age (Fig. 3a&b and Additional file 2: Figure S2). Of note, the expression of gene signatures for the trachea, salivary glands, and lungs increased while those for the cardiac myocytes and uterus decreased steadily throughout gestation; yet, more complex patterns emerged for the pituitary gland and fetal liver, whose expression signatures peaked and bottomed toward the end of the midtrimester, respectively (Fig. 3b).

##### Single-cell RNA-Seq signature analysis

**Fig. 4:**
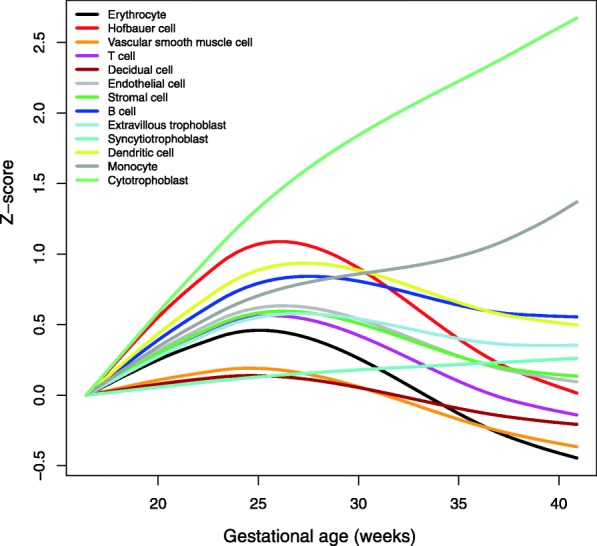
Changes in the expression of RNA Seq single-cell signatures with gestational age. For each single-cell signature, the expression of member genes (based on Tsang et al. [105]) was transformed into Z-scores and averaged in each AF sample. A Robust Locally Weighted Regression and Smoothing Scatterplots (LOESS) model fit through the Z-scores as a function of gestational age is shown using lines (see Fig. S3 for individual values). Single-cell signature trends are set to have the same value at 16 weeks of gestation. AF, amniotic fluid

The placenta emerged as one of the organs associated with both increase (Odds Ratio, OR = 2.1) (Additional file 11: Table S5) and decrease (OR = 3.1) (Additional file 12: Table S6) in gene expression from midtrimester to term gestation. We sought to further dissect placenta-specific gene expression by averaging the expression of genes specific to sub-populations of cells based on single-cell genomics studies. Among the 13 cell types identified by an RNA-Seq analysis of the placenta by Tsang et al. [105], the cytotrophoblast, monocyte, and syncytiotrophoblast expression increased monotonically from 16 weeks of gestation until term. By contrast, the Hofbauer cells, erythrocytes, vascular smooth muscle cells, B cells, T cells, and others showed more complex patterns (increase followed by decrease) (q < 0.05) (Fig. 4**,** Additional file 3: Figure S3). Of these single-cell signatures, that of the cytotrophoblast, defined as the average expression of the *FAM3B*, *FOXO4*, and *MIR205HG* genes, was the most highly modulated, being increased at term 1.8 SD relative to the midtrimester group, mostly due to the contribution of *FAM3B* gene expression.

### Differential splicing

**Fig. 5:**
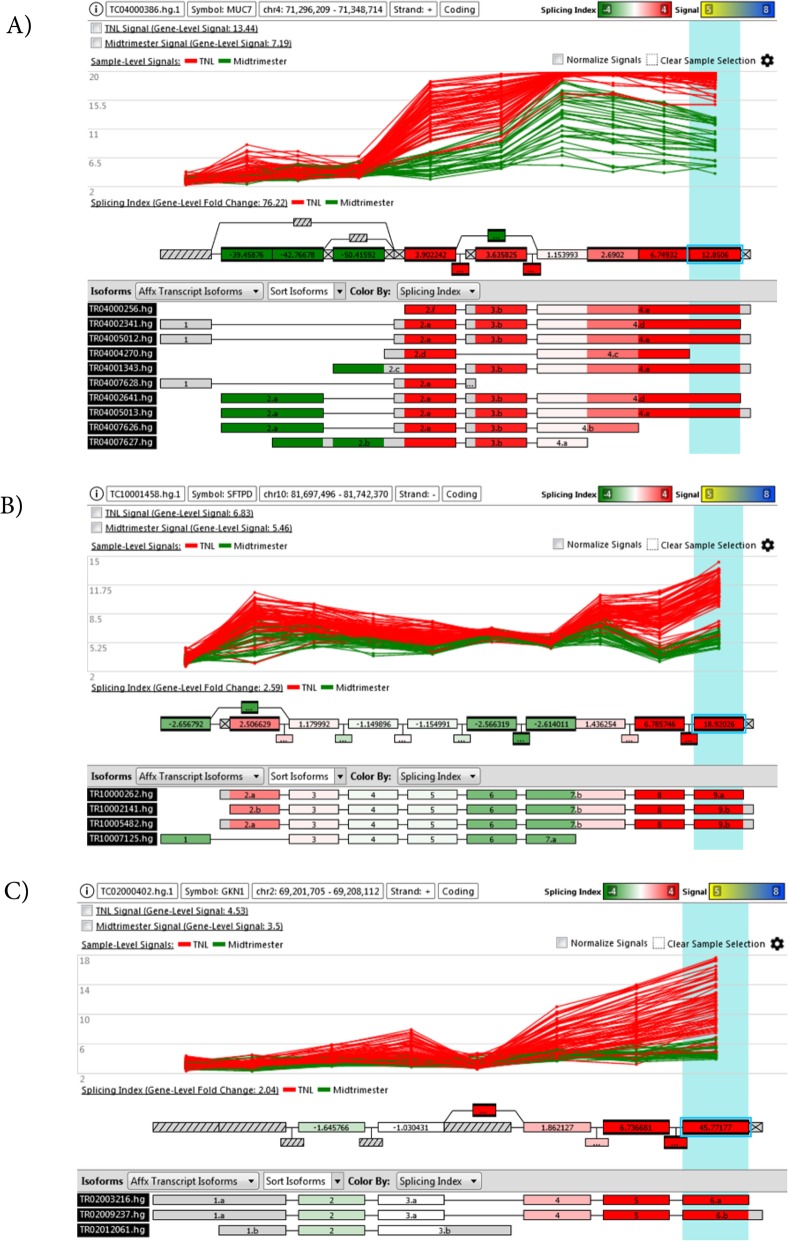
Example of differential expression and splicing associated with gestational age differences between midtrimester and term gestation groups. Each panel refers to a different gene (**a**: *MUC7*; **b**: *SFTPD*; **c**: *GKN1*). The top panel shows the normalized gene expression levels in each sample (line) and each probeset (dot) of a given patient. The middle panel shows a representation of the gene model with the color scale giving the splicing index for each probeset. The bottom layer shows possible transcript isoforms

Alternative splicing (AS) events associated with advancement from midtrimester to term gestation were identified in 17.5% (8566/48,820) of the genes detected and met the conditions for testing (see Methods) (exon q-value < 0.05 and SI > 2.0 or SI < − 2.0) (Additional file 13: Table S7). Of all tested genes, 25% of the coding genes and 4% of the non-coding genes displayed AS events associated with gestational-age difference from midtrimester to term gestation. Of note, 85% of all differential splicing results according to the SI method in TAC 4.0 software were also supported by the *diffSplice* algorithm (q < 0.05). Of approximately 54% of differentially spliced genes for which a particular type of AS event was identified by the Event Estimation algorithm, the most common types were the cassette exon (69%), alternative 5′ site (19%), alternative 3′ site (10%), and intron retention (2%) (Additional file 13: Table S7). For genes differentially expressed with gestational age, the AS analysis pinpointed the transcript isoforms likely responsible for overall gene expression changes: this is illustrated in Fig. 5 for the *MUC7* (salivary gland-specific), *SFTPD* (lung-specific), and *GKN1* (stomach-specific) genes. These genes were previously reported to be among those most differentially expressed with gestational age in AF based on 3′-end biased microarray platforms studies [120]. For example, our data suggest that most of the increase in expression of *MUC7* from midtrimester to term gestation can be explained by a short isoform of this gene (see transcript TR04000256, Fig. 5a).

**Fig. 6:**
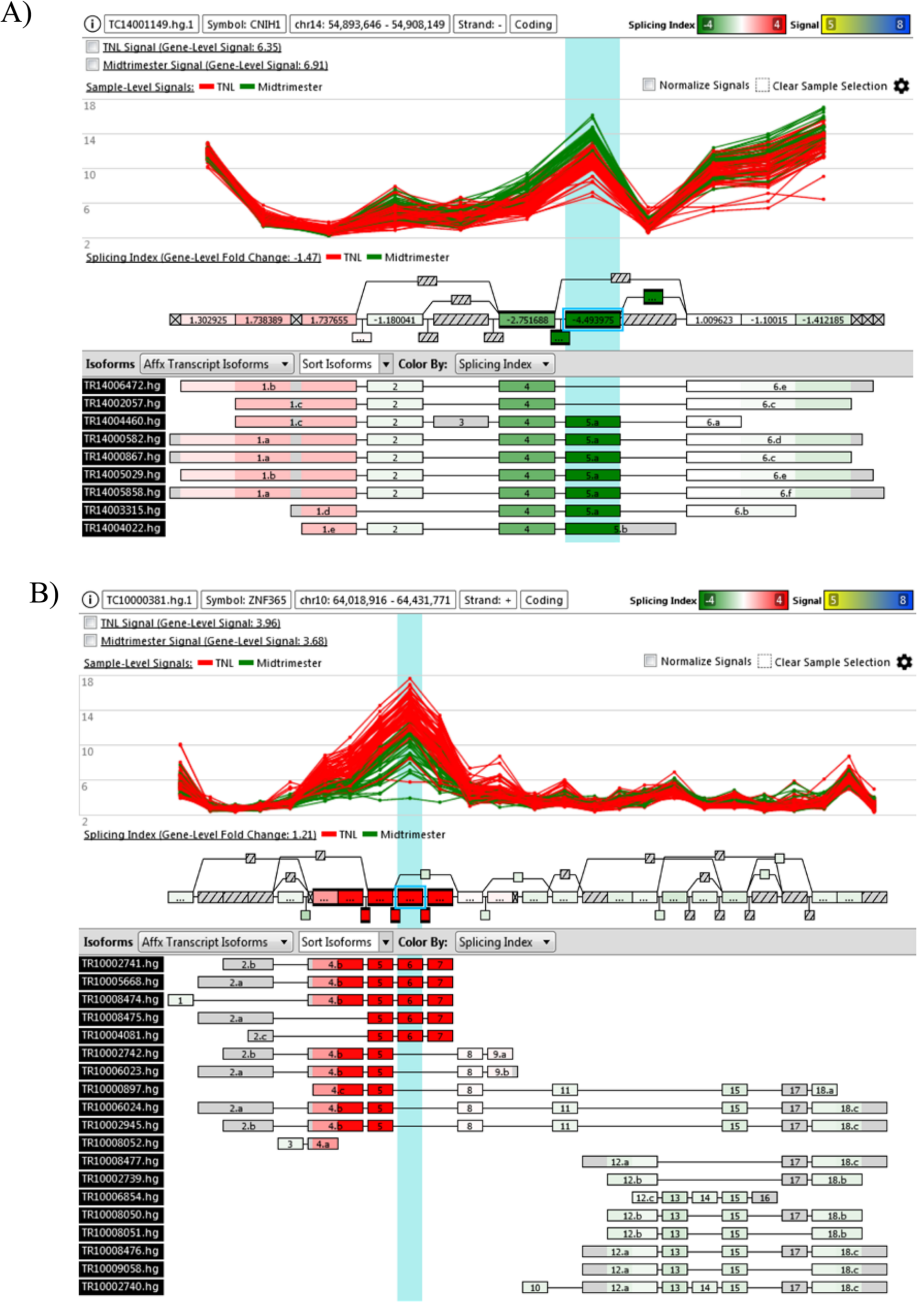
Example of differential splicing but not expression associated with gestational age differences between midtrimester and term groups. Each panel refers to a different gene (**a**: *CNIH1*; **b**: *ZNF365*). Details as shown in Fig. 3

The majority (76%) of differentially spliced genes were not differentially expressed, showcasing the importance of differential splicing in the study of the AF transcriptome. For instance, *CNIH1* skipped and *ZNF365* included a particular exon more frequently in the TNL group compared to the midtrimester group, yet neither gene met the criteria of being differentially expressed (Fig. 6). Functional profiling of differentially spliced genes identified about 800 DFLAT database biological processes as enriched that were not identified as such by analysis of differential expression, including 47 processes related to development (ear, central nervous system neurons, tongue, and spleen) as well as several immune-related processes (Additional file 14: Table S8). Among the MSigDB database pathways found to be enriched based on differential splicing, but not differential expression analysis, the REACTOME *adaptive immune system*, the BIOCARTA *MEF2D pathway*, and the KEGG *MAPK pathway* were among the most enriched (Additional file 15: Table S9). Tissues and cell types associated with gestational age by differential splicing analysis, but missed by differential expression analysis, were brain tissues (e.g. prefrontal cortex, globus pallidus, and cerebellum peduncles) and T cells (CD8+ and CD4+) (Table 3).

**Table 3:** Gene Atlas-based tissues and cell types associated with gestational-age differential splicing but not with differential expression from midtrimester to term gestation

Name	Count	Size	Odds Ratio	q
CD8+ T cells	101	206	2	0.000
CD4+ T cells	96	199	2	0.000
Prefrontal cortex	123	303	1.4	0.002
Whole brain	90	216	1.5	0.004
Cerebellum peduncles	54	120	1.7	0.004
Globus pallidus	33	67	2	0.005
Subthalamic nucleus	49	110	1.7	0.007
Pineal gland (night)	90	221	1.5	0.007
Uterus corpus	18	33	2.5	0.010
Cerebellum	44	102	1.6	0.019
Caudate nucleus	49	116	1.5	0.020
Parietal lobe	51	122	1.5	0.022
Pineal gland (day)	84	217	1.3	0.032
Occipital lobe	66	169	1.4	0.045
Pons	33	78	1.5	0.049

Count: number of differentially spliced genes associated with the ontology. Size: total number of genes associated with the ontology that were tested; odds ratio of enrichment based on a Fisher’s exact test; q: adjusted *p*-value

### Effect of fetal sex on the AF transcriptome

AF mRNA expression changes with fetal sex were found for 252 genes, with 215 being decreased and 37 being increased in expression in pregnancies with a male compared to those with a female fetus (Additional file 16: Table S10). All genes with increased expression in AF samples of women with a male fetus compared to those with a female fetus, were located on the chromosome Y (q < 0.05, OR = 64, Additional file 4: Figure S4), with ribosomal protein S4, Y-linked 1 (*RPS4Y1*) being the most increased in pregnancies with a male fetus (8-fold increase) (Additional file 16: Table S10). This gene was also reported by Larrabee et al. [83] as being present in the AF of women with a male fetus but not in those with a female fetus. Functional profiling analysis revealed that fetal sex gene expression differences were associated with one PID database pathway (*P 53 downstream pathway*) as well as in particular tissues (tongue and tonsils) and cell types (bronchial epithelial cells, CD71+ early erythroid cells) (all q < 0.05). When differential splicing associated with fetal sex was assessed, we found 240 significant genes, including some deemed also differentially expressed (e.g. *RPS4Y1*) and others that were not differentially expressed, including a transcript cluster for *TXLNGY* (Additional file 5: Figure S5). Chromosomes Y (odds ratio [OR] = 70) and X (OR = 2.4) were significantly enriched in genes with differential splicing between pregnancies with a male or a female fetus (q < 0.05).

### Effect of maternal characteristics and mode of sample collection

Motivated by the findings of Edlow et al. [99] regarding an association between the AF transcriptome and maternal obesity, we also compared gene expression between 38 obese (BMI > 30) and 33 lean (BMI < 25) women, while adjusting for fetal sex and gestational age at amniocentesis. No significant differences in expression and splicing were found with maternal obesity for individual genes. The same was true when limiting the analysis only to women sampled during the midtrimester, as in the original report by Edlow et al. [99] (Table 2). However, we found a weak but significant correlation of fold changes derived herein for the 182 genes reported to change with obesity by Edlow et al. [99] and present on the microarray platform herein (Spearman’s correlation 0.2, *p* = 0.02), with 62% of those genes matching in terms of the direction of change with obesity (Additional file 6: Figure S6).

Finally, we also examined the effect of other maternal characteristics, including ethnicity (African American versus Caucasian), parity (parous versus nulliparous), smoking status, as well as the mode of collection (cesarean delivery versus transabdominal amniocentesis) and indication for amniocentesis, while adjusting for variables that had a significant effect (gestational-age group and fetal sex). No significant differences in overall gene expression were found based on these analyses (all q > 0.1) (Table 2).

## Discussion

Amniotic fluid cfRNA analysis was proposed as a means to study real-time human fetal physiology and development [83, 85, 88, 99]. The results presented herein are in agreement with previous reports regarding differential gene expression from midtrimester to term gestation; yet, we have shown for the first time strong expression changes even during the midtrimester alone (before 21 weeks of gestation versus after). Tissue-specific mRNA expression patterns with gestation are found herein to be more complex than previously reported, owing to better coverage of the second-trimester gestational-age range. Leveraging previous single-cell genomics studies of the placenta, we also show for the first time that it is feasible to track signatures of placental single-cell populations by AF cfRNA analysis to assess the dynamic of crosstalk at the maternal-fetal interface. Finally, we present evidence that alternative splicing, a mechanism described to contribute to tissue-identity acquisition and organ development [123], is modulated in the AF with advancing gestational age and associated with the development of complex organ systems (e.g. brain).

### Amniotic fluid differential expression with advancing gestation

Hui et al. [98] and Kamath-Rayne et al. [85] reported that AF cfRNA displays dramatic changes with gestational age using 3′-end biased microarrays and RNA-Seq, respectively. Using a more recent microarray platform that probes both the coding and non-coding genes at exon-level resolution, we found 6194 differentially expressed transcript clusters (3447 unique ENTREZ database annotated genes) to be differentially expressed between midtrimester and term samples (Additional file 7: Table S1). The agreement between results presented herein and these two previous studies was high in terms of direction of change and correlation of fold changes (Fig. 2), yet the number of genes identified herein was larger owing to an increased sample size and sensitivity of the microarray platform employed. Enrichment analyses based on differentially expressed genes identified similar tissues and organ systems related to the *in utero* environment, fetal development, and preparation for life outside the uterus [83, 98]. However, given better coverage of the gestational-age span during midtrimester gestation, we show that the expression of tissue-specific signatures can have a complex pattern of modulation, including the fetal liver and cardiac myocytes, among others (Fig. 3**)**.

### Single-cell RNA-Seq signature modulation with advancing gestation

Single-cell RNA-seq signatures of populations of placental cells were recently described as a vocabulary to interpret the molecular crosstalk at the maternal-fetal interface [105, 106, 124]. The ability to track the expression of these signatures throughout gestation [125] and their alterations with obstetrical disease [105, 124] were also reported. The current study provides new and original evidence that fetal-specific (syncytiotrophoblast, cytotrophoblasts, Hofbauer cells, and vascular smooth muscle cells) or maternal-fetal origin cell populations (erythrocytes, monocytes, B cells and T cells) change with advancing gestation in AF. The increase in the expression of the monocyte and syncytiotrophoblast signatures reported in Fig. 4 is in agreement with observations based on cell-free [105] transcriptome analysis in maternal circulation. Nonetheless, the sharp increase during midtrimester gestation, followed by a decrease at near-term gestation, for Hofbauer and other cell signatures shown in Fig. 3 are for the first time described herein. Importantly, the AF single-cell RNA signatures of monocytes, T cells, and B cells mirrored the abundance of these immune cells throughout normal pregnancy as quantified by flow cytometry [78].

### Amniotic fluid differential splicing with advancing gestation

Through analysis of gene expression at the level of individual exons and exon-exon junctions, we identified AS events associated with gestational age and fetal sex. The exon junction arrays used in this study were previously compared to RNA-Seq for the purpose of differential splicing and found to have higher power when quantifying low-abundance transcripts as well as long non-coding RNAs that tend to be shorter than protein-coding gene counterparts [126]. Of interest, we found more genes (17.5% of the genes detected) displaying differential splicing than differential expression (10%) with gestational age. This can be explained, in part, by the fact that there is a higher threshold to claim differential expression than differential splicing. For differential expression, changes need to concur for multiple exonic regions (Fig. 5), while for differential splicing, changes for even a single or a few exonic region are sufficient (Fig. 6).

Genes showing differential splicing were associated with processes and pathways related to development and immunity and were specific to certain complex organ systems that were not identified as enriched based on differential expression analysis alone (Table 3). AS is recognized as a fundamental process by which cells expand their transcriptomic diversity, and it is particularly widespread in the nervous system [127]. Therefore, consideration of AS, as a means to assess the maturity or developmental stage of fetal organs, is important.

### Strengths and limitations

This is the largest study of the AF transcriptome in which coding and non-coding gene expression was profiled at exon-level resolution in approximately 100 normal pregnancies. One of the strengths of the study is the simultaneous assessment of the effect of genetic (fetal sex and maternal ethnicity), physiologic (gestational age, parity), and maternal risk factors (smoking, obesity) on the transcriptome. Also, the observation that there are no significant effects related to the AF sample collection mode (cesarean delivery versus transabdominal amniocentesis) is an important addition to the literature. This suggests that samples collected by both modalities can be used to establish a gene expression reference. Although the sample size for this particular comparison (cesarean delivery versus transabdominal amniocentesis at term) was low, the reduced magnitude of such an effect was reassuring. Of note, the comparison between the two types of sample collection at term gestation was also cofounded by the indication for amniocentesis, since transabdominal collection was performed to assess fetal lung maturity while collection during cesarean delivery was done for research purposes. The use of multiple types of functional profiling approaches and single-cell signatures as a means to interpret differential expression and splicing results is also a strength. Finally, although no additional wet-lab confirmatory results were presented, the *in silico* validation of overall gene expression differences with gestational age and fetal sex, through correlations with previous reports, increases confidence in the novel results presented herein, such as differential splicing with advancing gestation and fetal gender. Among the limitations, we would also note a lower statistical power for some of the sub-analyses assessing the effects of maternal race and obesity in midtrimester samples.

## Conclusions

We reported herein the largest AF cell-free transcriptomics study that catalogues physiologic adaptations with advancing gestation in normal pregnancy and surveys the effects of relevant maternal, fetal, and experimental covariates on the transcriptome. Our data show that AF mRNA profiles can be used to track placental function through single-cell specific signatures, as a readout of the maternal-fetal crosstalk during pregnancy. We also propose that alternative splicing evaluation should be a part of the future development of fetal testing to assess organ maturity; this information could be used to inform clinical management given the current debate about the usefulness of fetal lung maturity evaluation.

## Supplementary information

**Additional file 1: Figure S1.** Representation of chromosome-specific differential expression between midtrimester and term gestation groups. The outer circle shows the chromosomes, with significant enrichment being marked with * (q < 0.05). The inner circle shows the log_2_ fold change (term/midtrimester) of differentially expressed genes, positioned based on their genomic coordinates within each chromosome. Blue denotes increase and red denotes decrease in the term group. Values greater than 3.0, in absolute value, were truncated to 3.0 to enhance display

**Additional file 2: Figure S2.** Changes in the expression of tissue-specific signatures with gestational age superposed to patient-specific values. For each tissue, the expression of the top 20 most-specific genes (based on the Gene Atlas dataset) was transformed into a Z-score and averaged in each AF sample (dots). A Robust Locally Weighted Regression and Smoothing Scatterplots (LOESS) model fit through the Z-scores as a function of gestational age is shown using lines. All differentially expressed tissue signatures (term versus midtrimester or linear correlation within the midtrimester group) are shown. AF, amniotic fluid

**Additional file 3: Figure S3.** Changes in the expression of RNA Seq single-cell signatures with gestational age. For each single-cell signature, the expression the all specific genes (based on Tsang et al. [105]) was transformed into a Z-score and averaged in each AF sample (dots). A Robust Locally Weighted Regression and Smoothing Scatterplots (LOESS) model fit through the Z-scores as a function of gestational age is shown using lines. AF, amniotic fluid

**Additional file 4: Figure S4.** Representation of chromosome-specific differential expression between pregnancies with a male those with a female fetus. The outer circle shows the chromosomes with significant enrichment being marked with * (q < 0.05). The inner circle shows the log_2_ fold change (male/female) of differentially expressed genes, positioned based on their genomic coordinates within each chromosome. Blue denotes increase and red denotes decrease in the males. Values greater than 3.0 in absolute value were truncated to 3.0

**Additional file 5: Figure S5.** Example of differential splicing with fetal sex for TXLNGY gene. Details as shown in Fig. 5

**Additional file 6: Figure S6.** Correlation of expression changes with maternal obesity between studies. Each dot represents a unique annotated gene. The y axis represents the log_2_ fold change (obese/lean) obtained in the current study. The x-axis represents the log_2_ expression of 182 genes detected as present in the current study among those reported as differentially expressed with obesity by Edlow et al. [99]. R: Spearman’s correlation coefficient

**Additional file 7: Table S1.** Genes differentially expressed with advancing gestational age from midtrimester to term. The table includes the Affymetrix transcript cluster identifier (ID), gene symbol, gene name, ENTREZ database identifier, chromosome and strand information, genomic coordinates (start and stop), number of microarray probes used to measure the gene expression, assignment to coding or non-coding region, log_2_ expression change (term/midtrimester), nominal *p*-value, and adjusted *p*-value (q-value)

**Additional file 8: Table S2.** Genes differentially expressed with gestational age during midtrimester. Columns are the same as in Table S1, except that the comparison is between late (21.1–24.0 weeks) versus early (16.4–21.0 weeks) midtrimester gestation

**Additional file 9: Table S3.** Biological processes from DFLAT database associated with gestational age differential expression from midtrimester to term. Count: number of differentially expressed genes associated with the ontology. Size: total number of genes associated with the ontology that were tested; odds ratio of enrichment based on a Fisher’s exact test; q: adjusted *p*-value

**Additional file 10: Table S4.** MSigDB database canonical pathways associated with gestational age differential expression from midtrimester to term. Columns are as shown in the Table S3 legend

**Additional file 11: Table S5.** Gene Atlas-based tissues and cell types associated with increased expression in term vs midtrimester samples. Columns are as shown in the Table S3 legend

**Additional file 12: Table S6.** Gene Atlas-based tissues and cell types associated with increased expression in midtrimester vs term samples. Columns are as shown in the Table S3 legend

**Additional file 13: Table S7.** Genes differentially spliced with gestational age (term versus midtrimester). The table includes results for the single most-significant exon or exon junction based on splicing index analysis. The columns include the Affymetrix transcript cluster identifier (ID), gene symbol, gene name, probeset identifier, exon splicing index, corresponding p-value, adjusted p-value, name of the alternative splicing event type, and adjusted p-value based on the diffSplice algorithm

**Additional file 14: Table S8.** Biological processes from DFLAT database associated with gestational-age differential splicing but not with differential expression from midtrimester to term. Columns are as shown in the Table S3 legend

**Additional file 15: Table S9.** MSigDB database canonical pathways associated with gestational-age differential splicing but not with differential expression from midtrimester to term. Columns are as shown in the Table S3 legend

**Additional file 16: Table S10.** Genes differentially expressed with fetal sex. Columns are the same as in Table S1, except that the comparison is between pregnancies with a male versus a female fetus

## Data Availability

The raw and processed gene expression data and relevant patient characteristics were deposited in the Gene Expression Omnibus (https://www.ncbi.nlm.nih.gov/geo/query/acc.cgi?acc=GSE133824).
