# Supplementary figures and images for: Amniotic fluid cell-free transcriptome: a glimpse into fetal development and placental cellular dynamics during normal pregnancy
Source: BMC Med Genomics. 2020 Feb 12;13:25. doi: 10.1186/s12920-020-0690-5 (PMC7017452; doi:10.1186/s12920-020-0690-5)

**Figure S1**

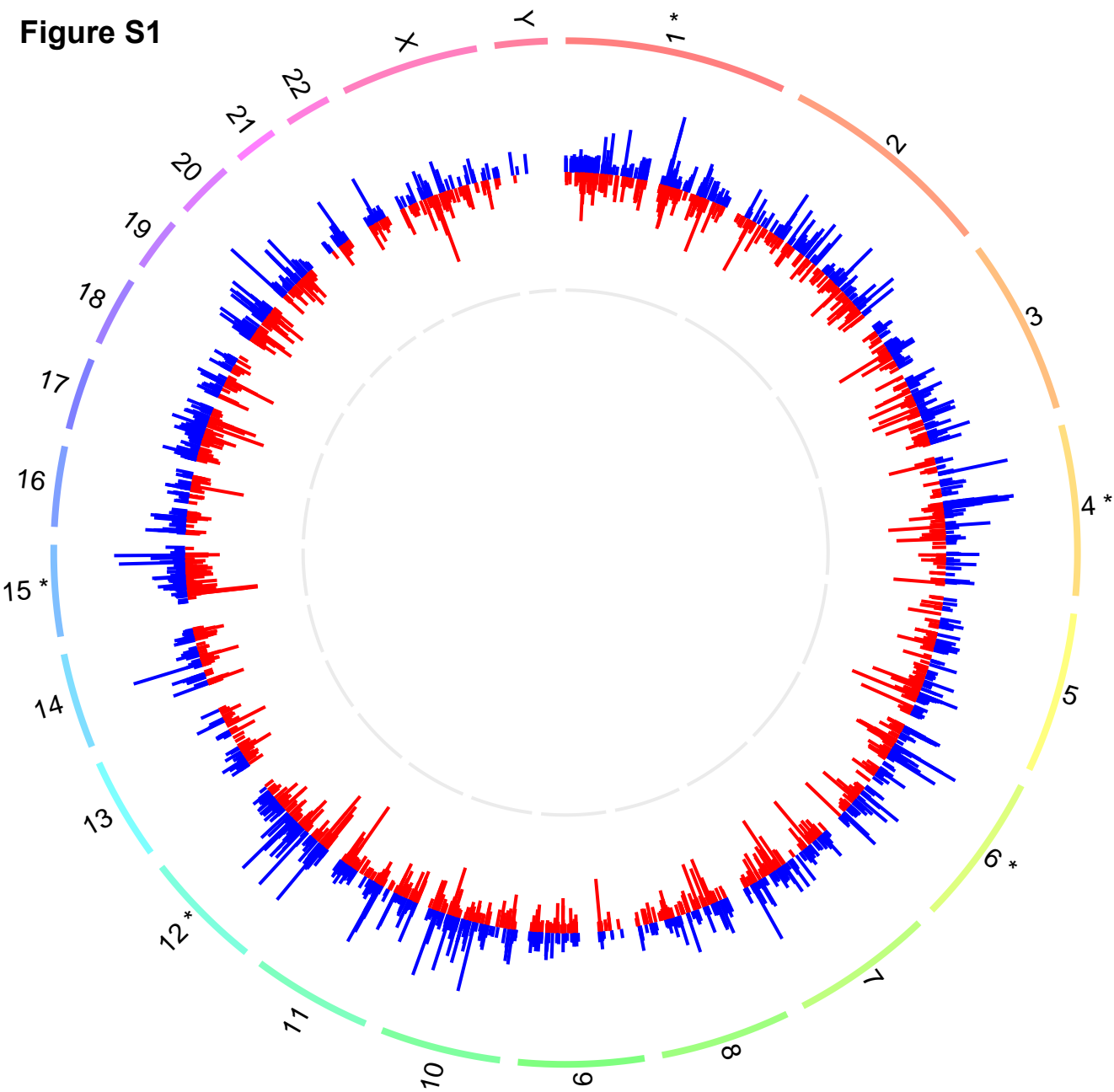

Supplement: Supplementary file 1 — Additional file 1: Figure S1. Representation of chromosome-specific differential expression between midtrimester and term gestation groups. The outer circle shows the chromosomes, with significant enrichment being marked with * (q < 0.05). The inner circle shows the log2 fold change (term/midtrimester) of differentially expressed genes, positioned based on their genomic coordinates within each chromosome. Blue denotes increase and red denotes decrease in the term group. Values greater than 3.0, in absolute value, were truncated to 3.0 to enhance display [file 12920_2020_690_MOESM1_ESM.pdf]

**Figure S2**

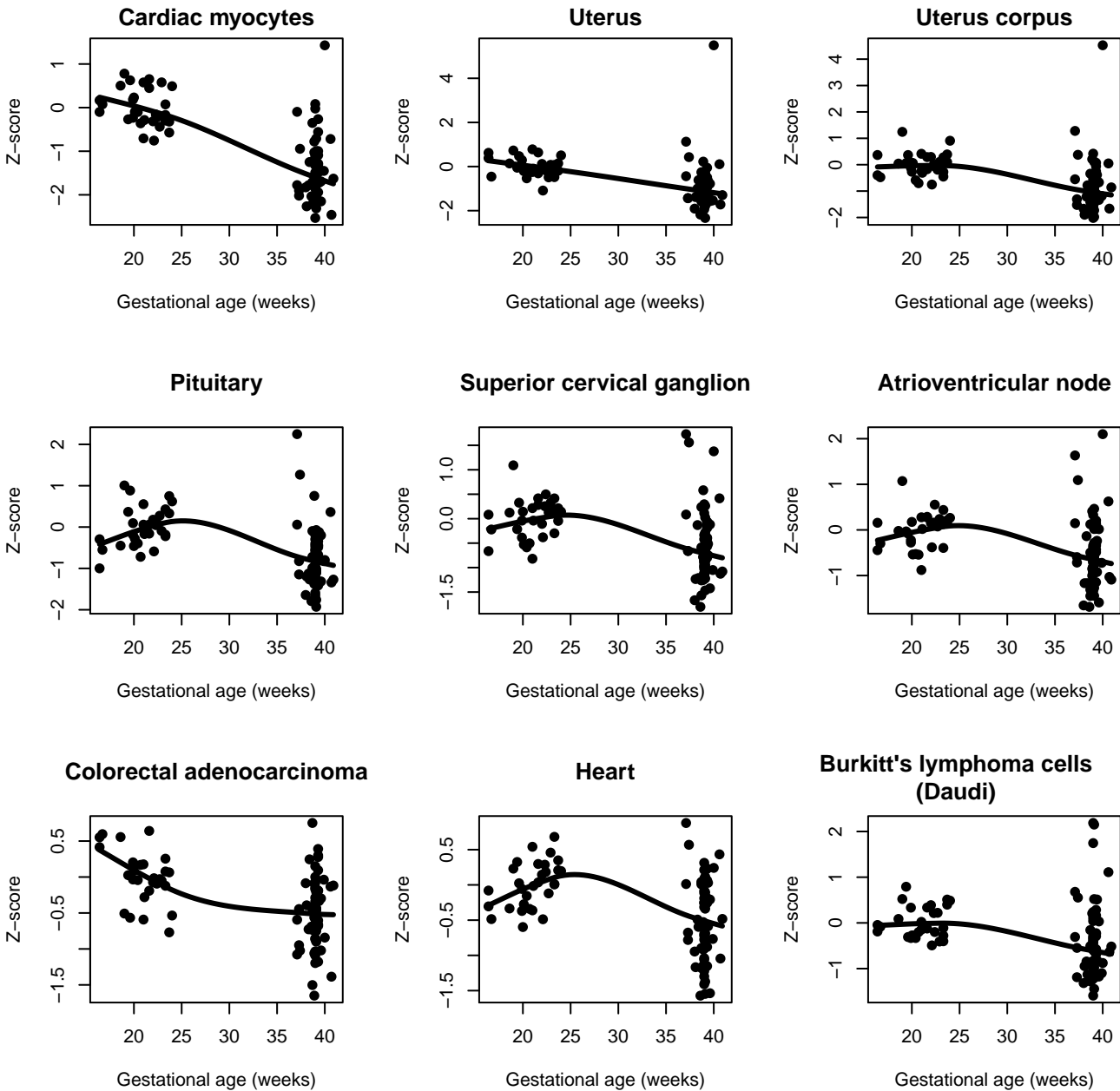

**Figure S2**

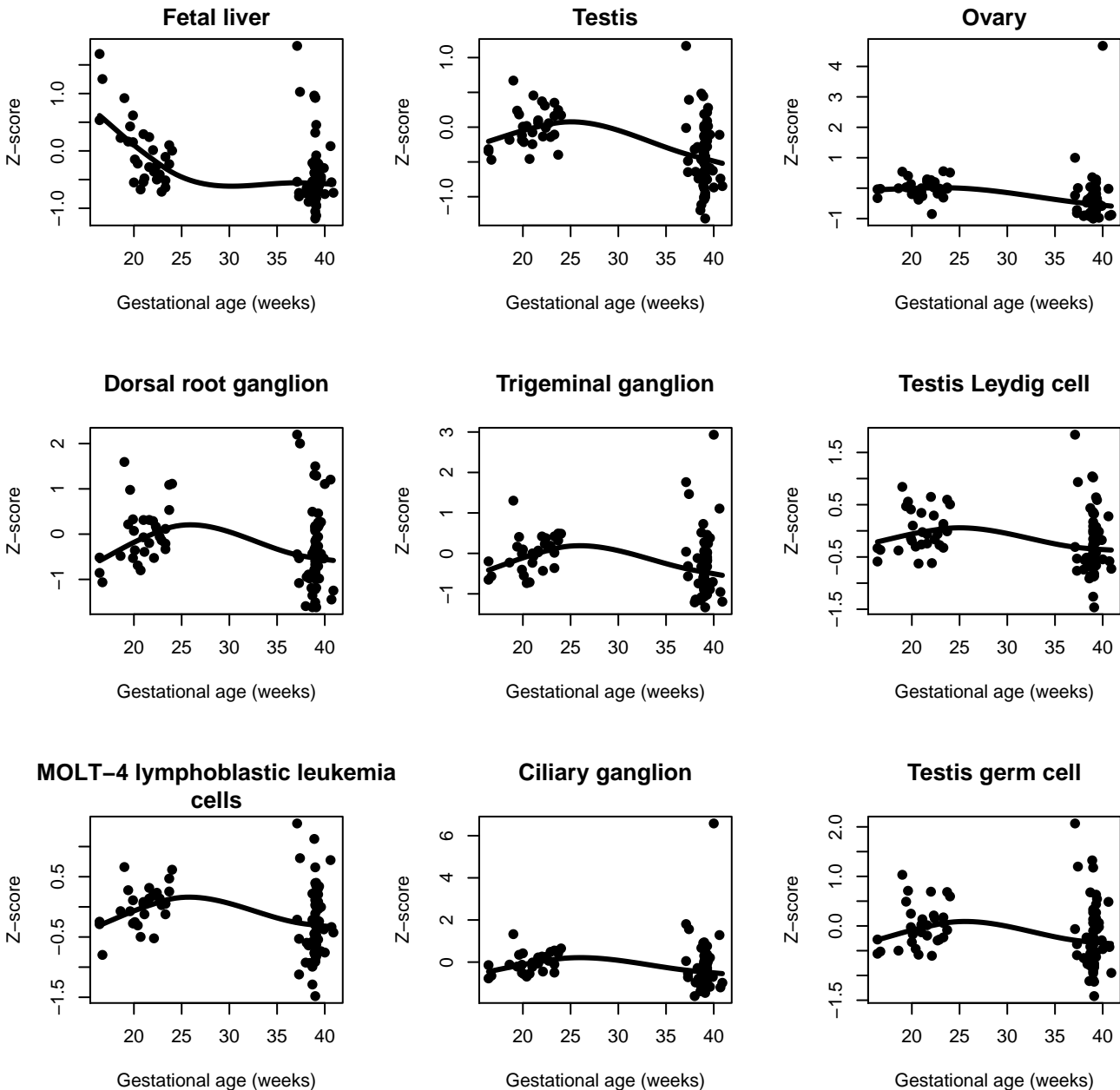

**Figure S2**

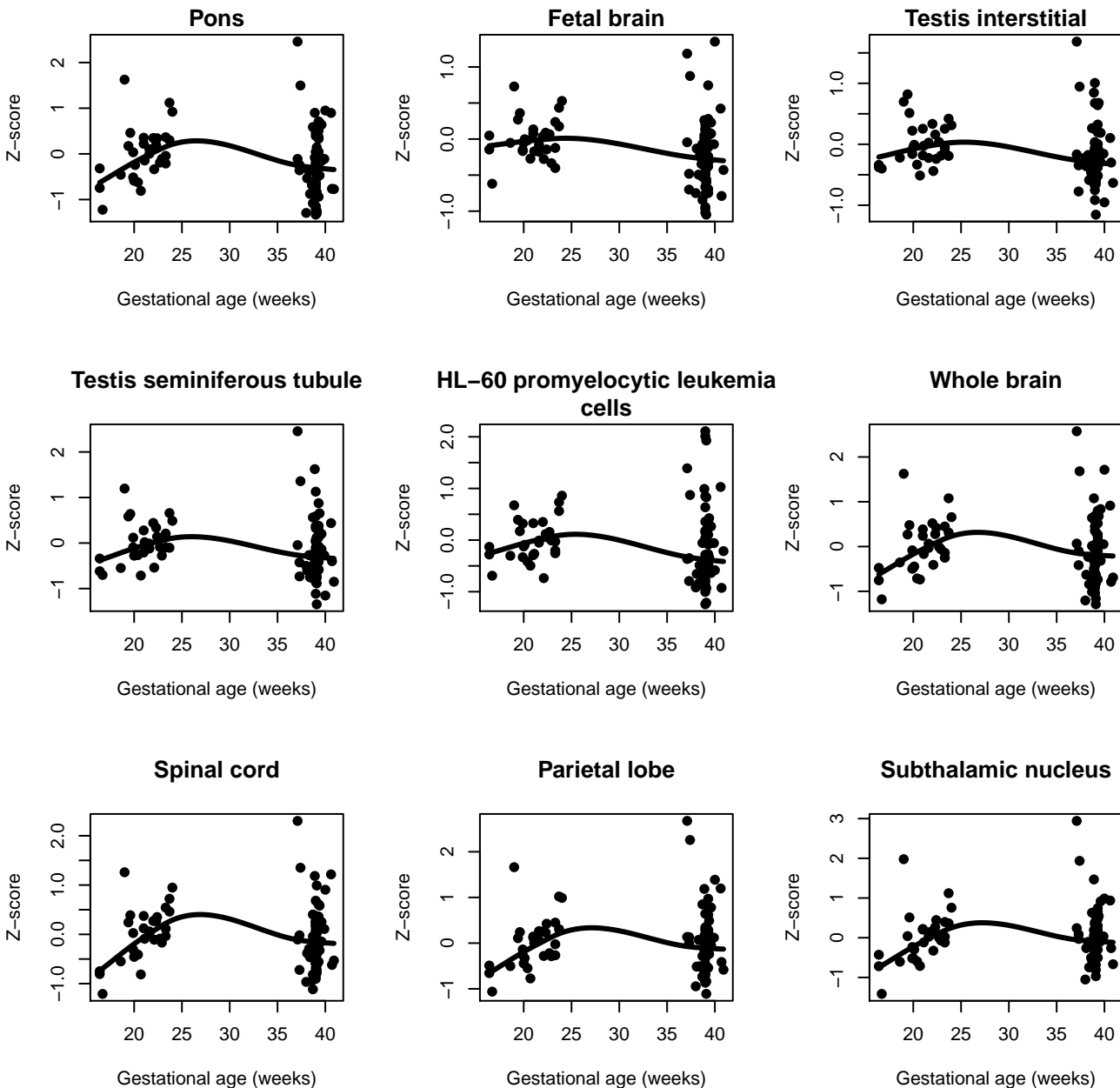

**Figure S2**

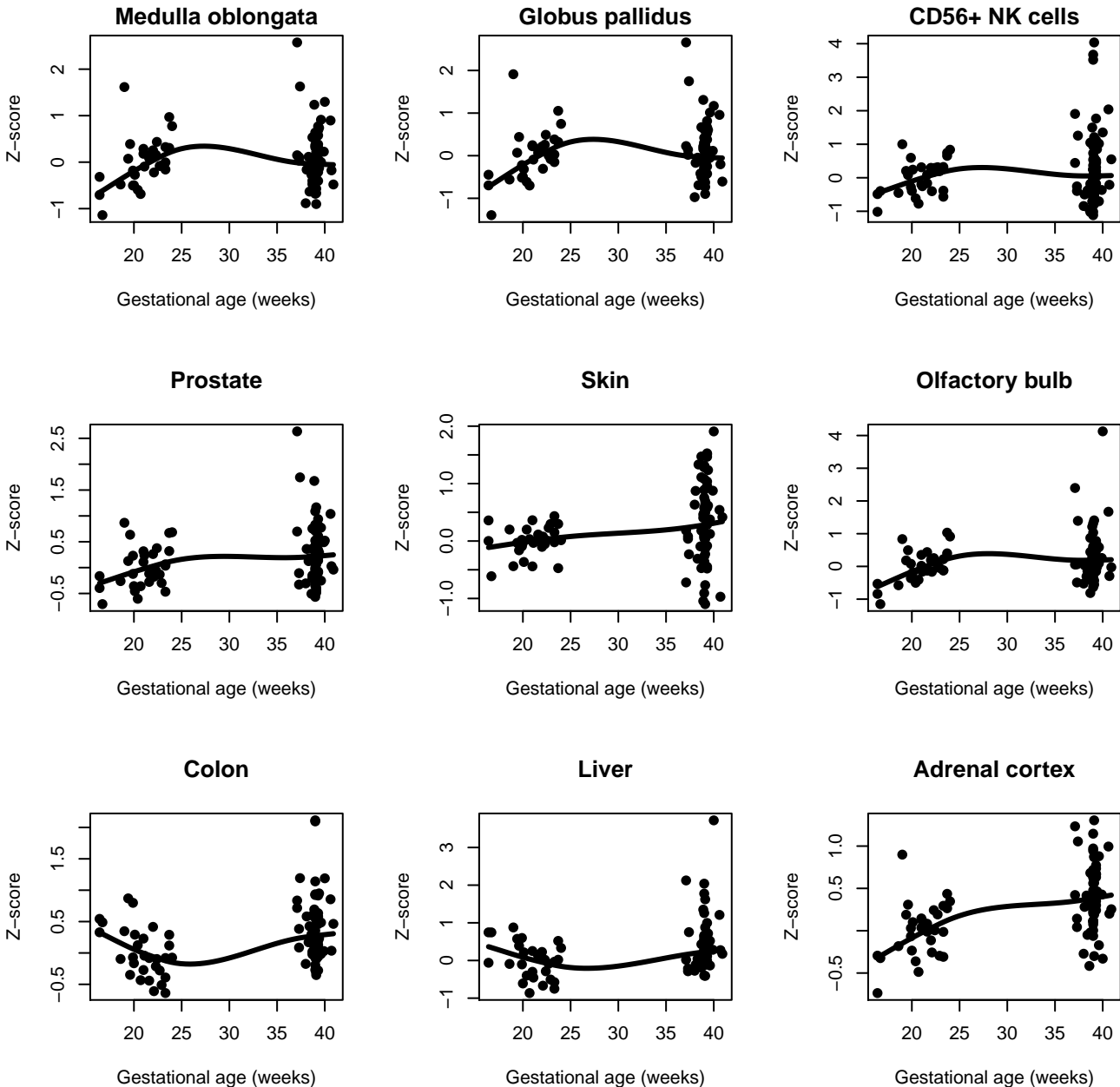

**Figure S2**

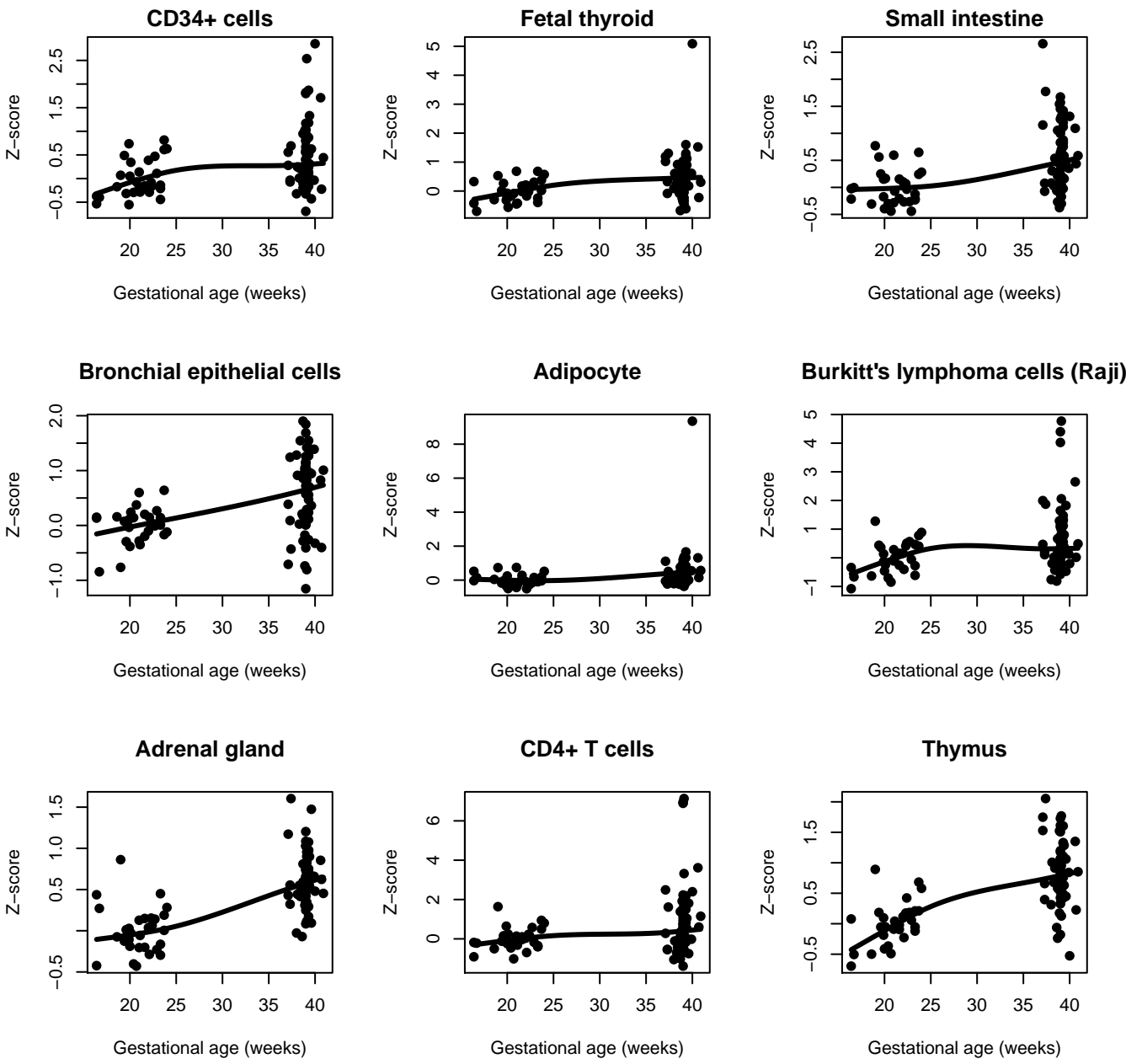

**Figure S2**

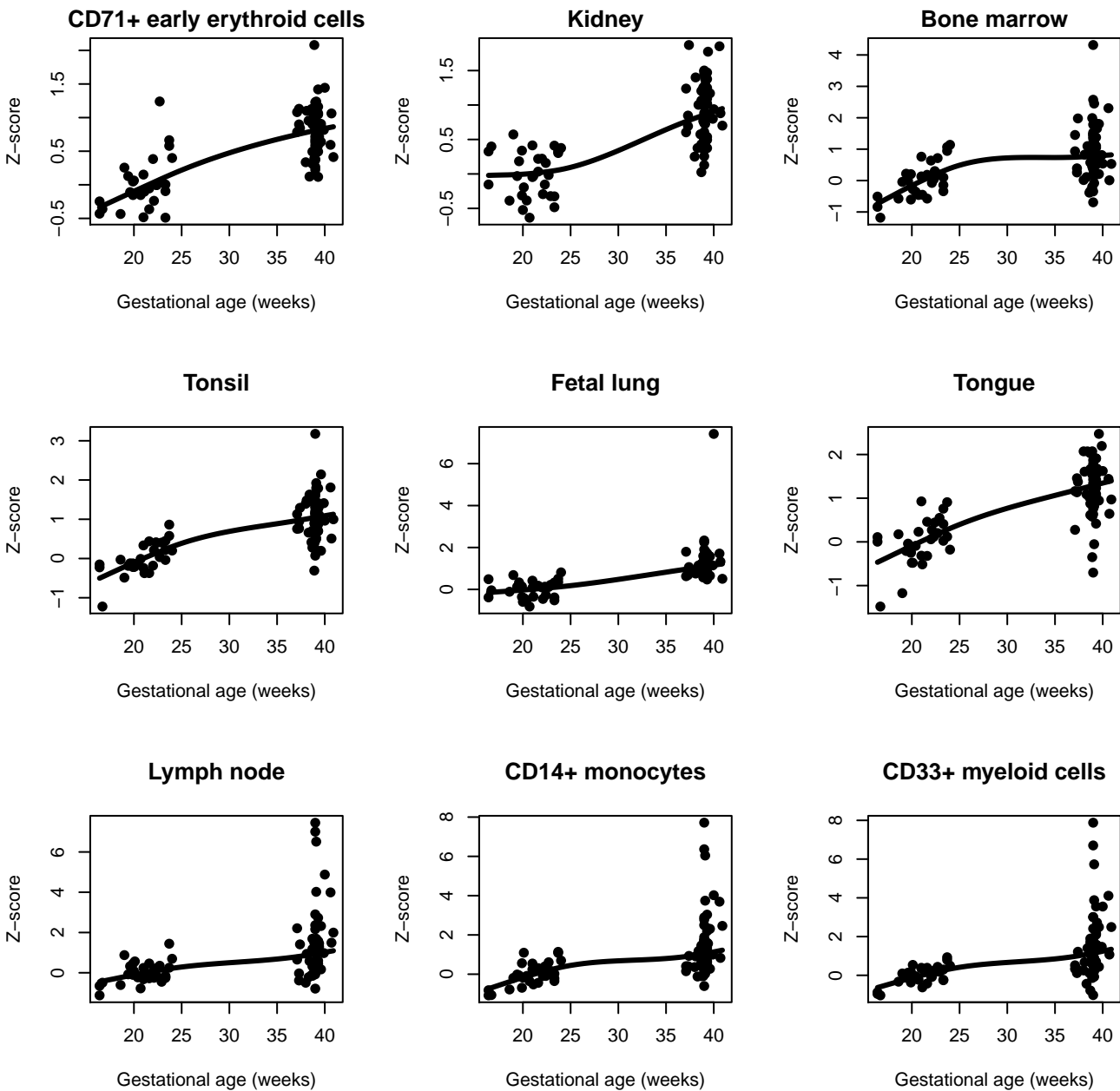

**Figure S2**

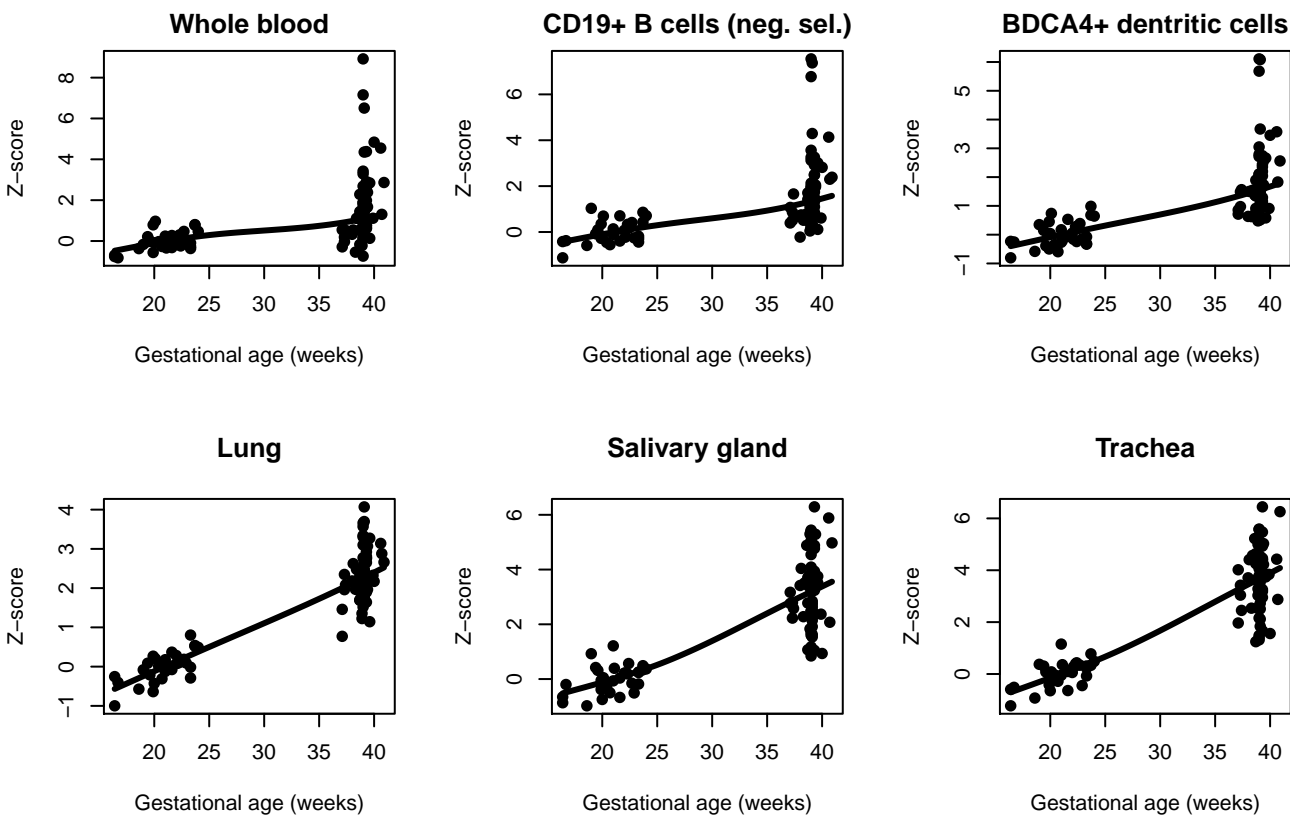

Supplement: Supplementary file 2 — Additional file 2: Figure S2. Changes in the expression of tissue-specific signatures with gestational age superposed to patient-specific values. For each tissue, the expression of the top 20 most-specific genes (based on the Gene Atlas dataset) was transformed into a Z-score and averaged in each AF sample (dots). A Robust Locally Weighted Regression and Smoothing Scatterplots (LOESS) model fit through the Z-scores as a function of gestational age is shown using lines. All differentially expressed tissue signatures (term versus midtrimester or linear correlation within the midtrimester group) are shown. AF, amniotic fluid [file 12920_2020_690_MOESM2_ESM.pdf]

**Figure S3**

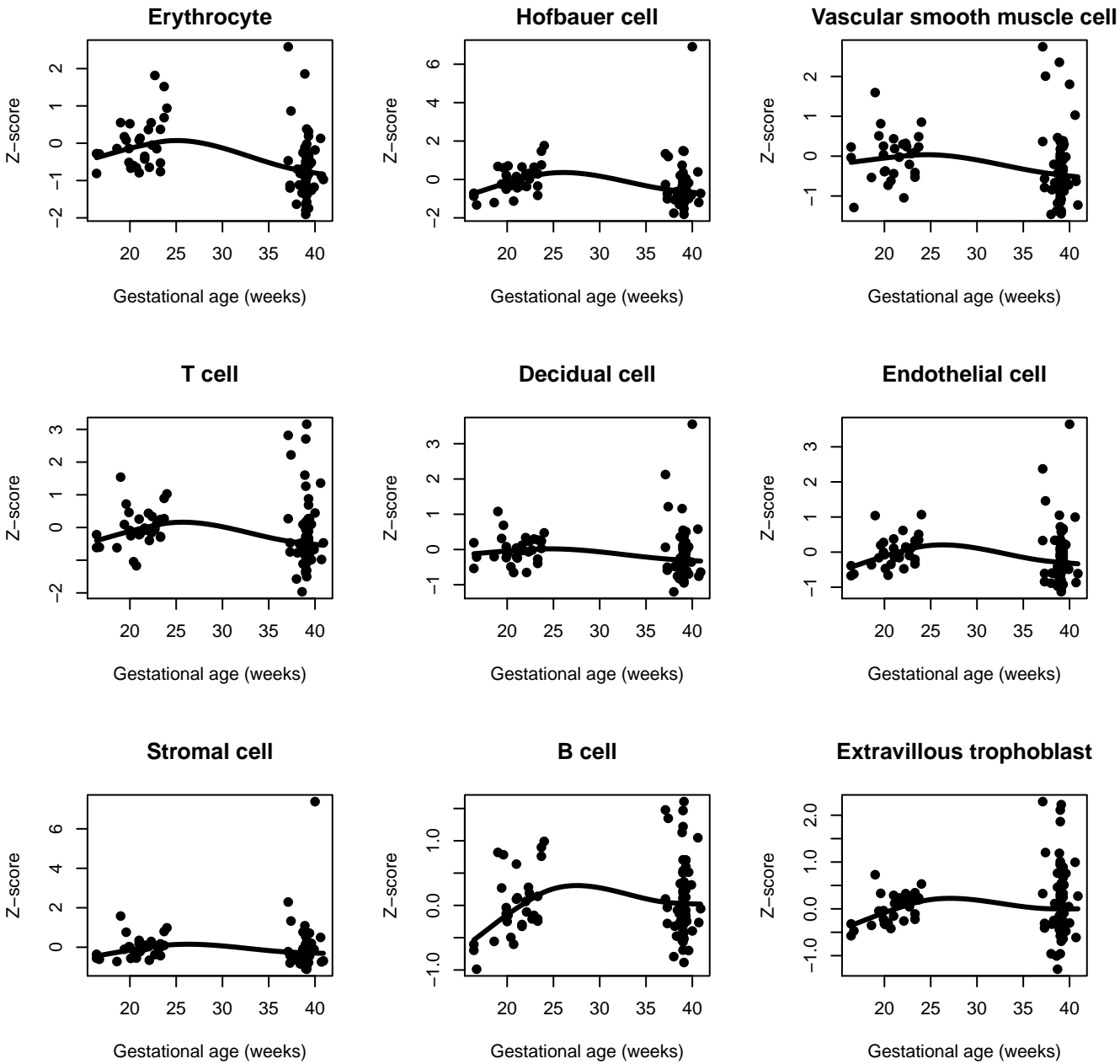

**Figure S3**

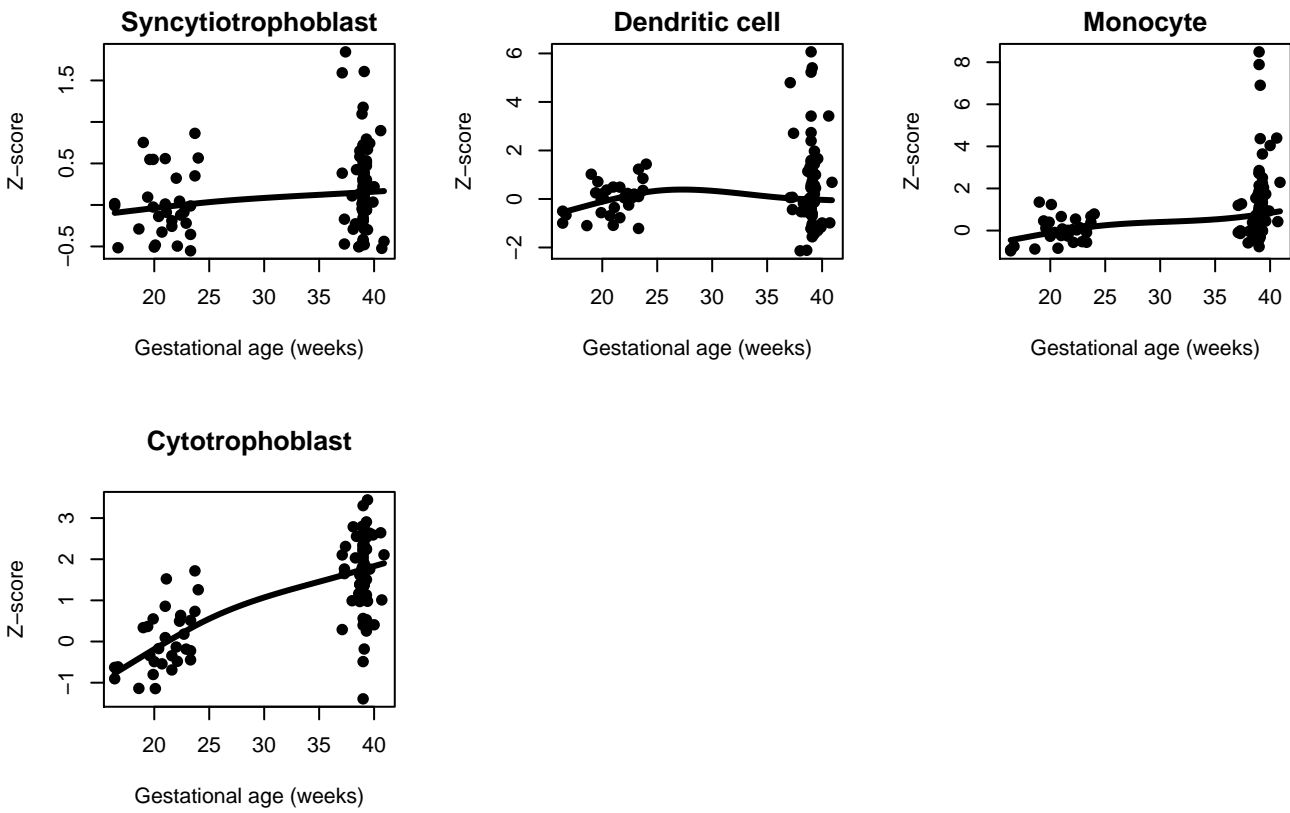

Supplement: Supplementary file 3 — Additional file 3: Figure S3. Changes in the expression of RNA Seq single-cell signatures with gestational age. For each single-cell signature, the expression the all specific genes (based on Tsang et al. [105]) was transformed into a Z-score and averaged in each AF sample (dots). A Robust Locally Weighted Regression and Smoothing Scatterplots (LOESS) model fit through the Z-scores as a function of gestational age is shown using lines. AF, amniotic fluid [file 12920_2020_690_MOESM3_ESM.pdf]

## Figure S4

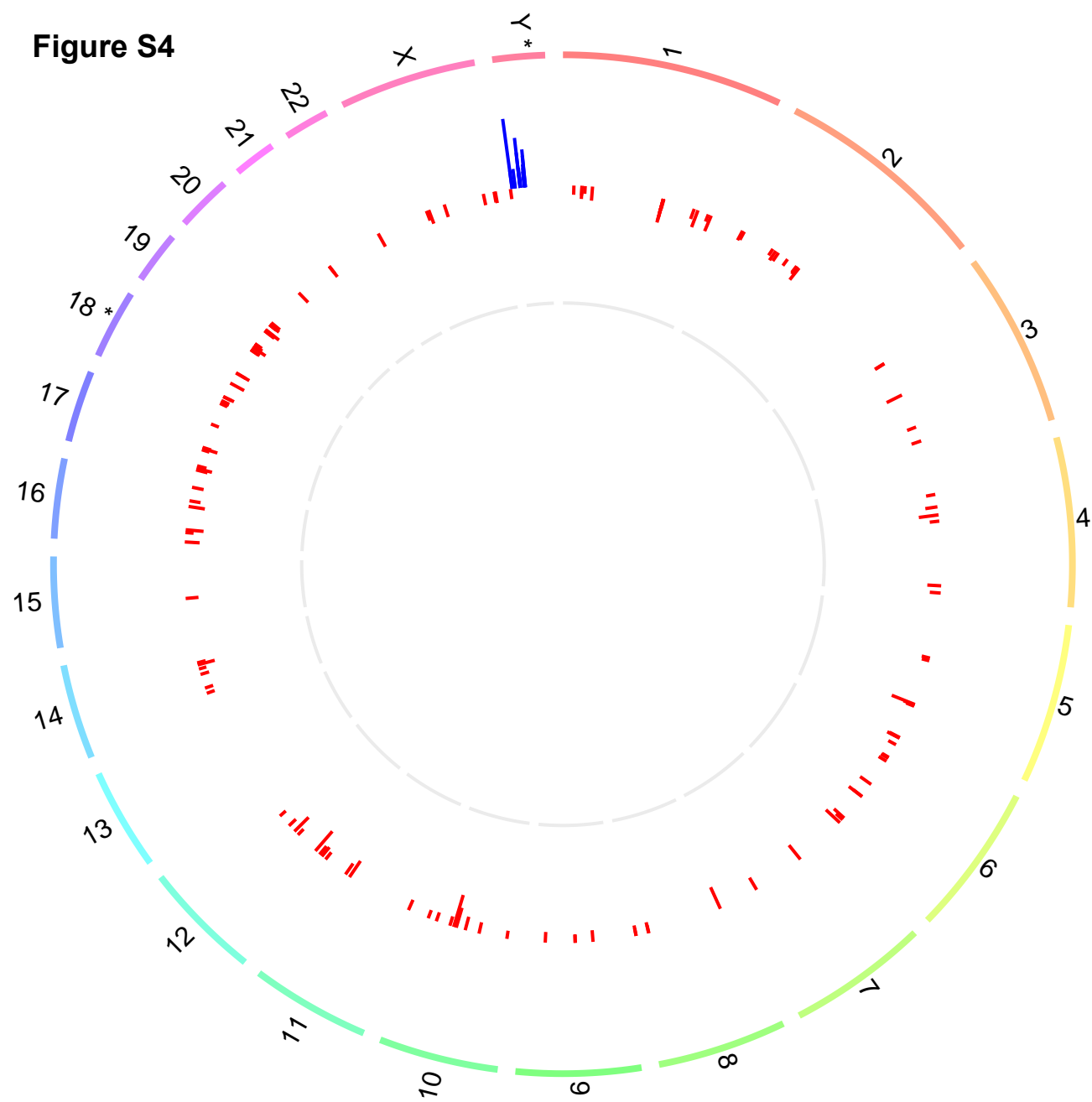

Supplement: Supplementary file 4 — Additional file 4: Figure S4. Representation of chromosome-specific differential expression between pregnancies with a male those with a female fetus. The outer circle shows the chromosomes with significant enrichment being marked with * (q < 0.05). The inner circle shows the log2 fold change (male/female) of differentially expressed genes, positioned based on their genomic coordinates within each chromosome. Blue denotes increase and red denotes decrease in the males. Values greater than 3.0 in absolute value were truncated to 3.0 [file 12920_2020_690_MOESM4_ESM.pdf]

Figure S5

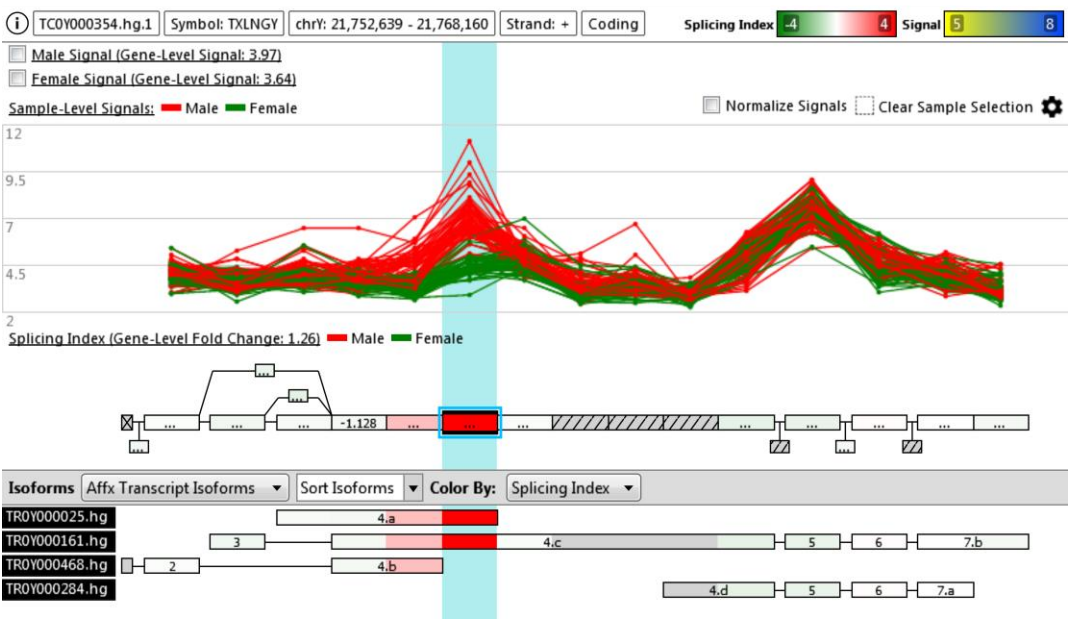

Supplement: Supplementary file 5 — Additional file 5: Figure S5. Example of differential splicing with fetal sex for TXLNGY gene. Details as shown in Fig. 5 [file 12920_2020_690_MOESM5_ESM.pdf]

Figure S6

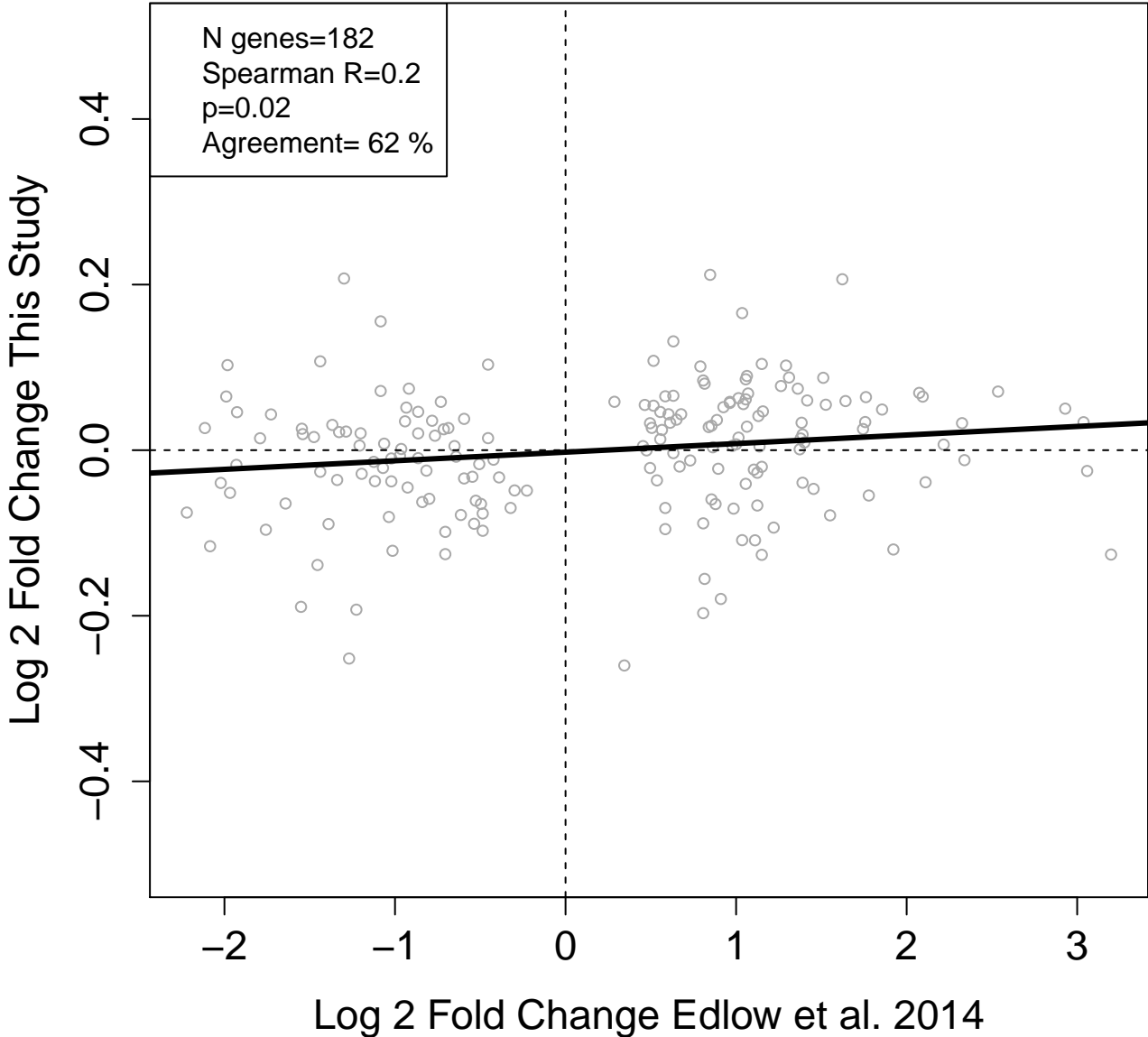

Supplement: Supplementary file 6 — Additional file 6: Figure S6. Correlation of expression changes with maternal obesity between studies. Each dot represents a unique annotated gene. The y axis represents the log2 fold change (obese/lean) obtained in the current study. The x-axis represents the log2 expression of 182 genes detected as present in the current study among those reported as differentially expressed with obesity by Edlow et al. [99]. R: Spearman’s correlation coefficient [file 12920_2020_690_MOESM6_ESM.pdf]
